# Fe_3_O_4_@nano-cellulose/Cu(ii): a bio-based and magnetically recoverable nano-catalyst for the synthesis of 4*H*-pyrimido[2,1-*b*]benzothiazole derivatives†

**DOI:** 10.1039/c8ra09203f

**Published:** 2019-01-11

**Authors:** Nasrin Safajoo, Bi Bi Fatemah Mirjalili, Abdolhamid Bamoniri

**Affiliations:** Department of Chemistry, College of Science, Yazd University Yazd P. O. Box 89195-741 Islamic Republic of Iran fmirjalili@yazd.ac.ir +98 3538210644 +98 3531232672; Department of Organic Chemistry, Faculty of Chemistry, University of Kashan Kashan Islamic Republic of Iran

## Abstract

Fe_3_O_4_@nano-cellulose/Cu(ii) as a green bio-based magnetic catalyst was prepared through *in situ* co-precipitation of Fe^2+^ and Fe^3+^ ions in an aqueous suspension of nano-cellulose. The mentioned magnetically heterogeneous catalyst was characterized by FT-IR, XRD, VSM, FESEM, TEM, XRF, EDS and TGA. In this research, the synthesis of 4*H*-pyrimido[2,1-*b*]benzothiazole derivatives was developed *via* a three component reaction of aromatic aldehyde, 2-aminobenzothiazole and ethyl acetoacetate using Fe_3_O_4_@nano-cellulose/Cu(ii) under solvent-free condition at 80 °C. Some advantages of this protocol are good yields, environmentally benign, easy work-up and moderate reusability of the catalyst. The product structures were confirmed by FT-IR, ^1^H NMR, and ^13^C NMR spectra.

## Abstract 2

Fe_3_O_4_@nano-cellulose/Cu(ii) as a green bio-based magnetic catalyst are prepared, characterized and applied for synthesis of 4*H*-pyrimido[2,1-*b*]benzothiazoles with good to excellent yields.

## Graphical abstract

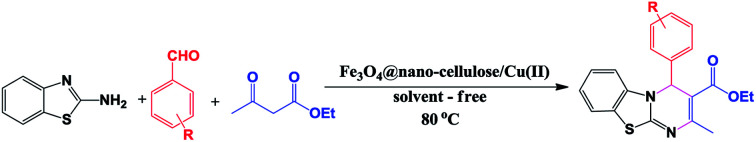

## Introduction

Fused heterocyclic compounds containing nitrogen and sulfur are important compounds because of their pharmacological properties.^1^ Among these compounds benzothiazoles and pyrimido[2,1-*b*]benzothiazoles have attracted considerable interest. Some of these compounds have various biological activities such as antiviral,^2,3^ antitumor,^4–6^ antiinflammatory,^7,8^ antiallergic,^9^ antimicrobial,^10,11^ anticonvulsant,^12^ antiproliferative^13^ and antifungal activities.^14^ Pyrimido[2,1-*b*]benzothiazole derivatives were synthesized through multicomponent reaction between 2-amino benzothiazole, aromatic aldehydes and β-ketoesters.^15–18^ Previously, this protocol has been catalyzed by iron fluoride,^19^ pyridine,^11^ acetic acid,^20^ 1,1,3,3-*N*,*N*,*N*′,*N*′-tetramethylguanidinium trifluoroacetate (TMGT),^16^ tetrabutylammonium hydrogen sulfate (TBAHS),^17^*N*-sulfonic acid modified poly(styrene-maleic anhydride) (SMI-SO_3_H),^21^ chitosan,^18^ aluminum trichloride^22^ and Fe_3_O_4_@nano-cellulose/TiCl.^23^ Some of the reported protocols have harsh conditions and long reaction times. Thus, in this work, a new simple protocol for the synthesis of these compounds is reported.

Biopolymers, especially cellulose and its derivatives, have some unparalleled properties, which make them attractive alternatives for ordinary organic or inorganic supports for catalytic applications.^24^ Cellulose is the most abundant natural material in the world and it can play an important role as a biocompatible, renewable resource and biodegradable polymer containing OH groups.^25^ Cotton is a natural, cheap, and readily available source of cellulose. Fe_3_O_4_ nanoparticles are coated with various materials such as surfactants,^26^ polymers,^27,28^ silica,^29^ cellulose^23^ and carbon^30^ to form core–shell structures. Magnetic nanoparticles as heterogeneous supports have many advantages such as high dispersion in reaction media and easy recovery by an external magnet.^31–38^ Cu(ii) as a safe and ecofriendly cation is a good Lewis acid and can activate the carbonyl group for nucleophilic addition reactions. Thus, the main purpose of the present work is the preparation of Fe_3_O_4_@nano-cellulose/Cu(ii) as a new and bio-based magnetic nanocatalyst for one-pot synthesis of pyrimido[2,1-*b*]benzothiazoles *via* condensation of aromatic aldehydes, ethyl acetoacetate and 2-aminobenzothiazole.

## Results and discussion

**Scheme 1:**
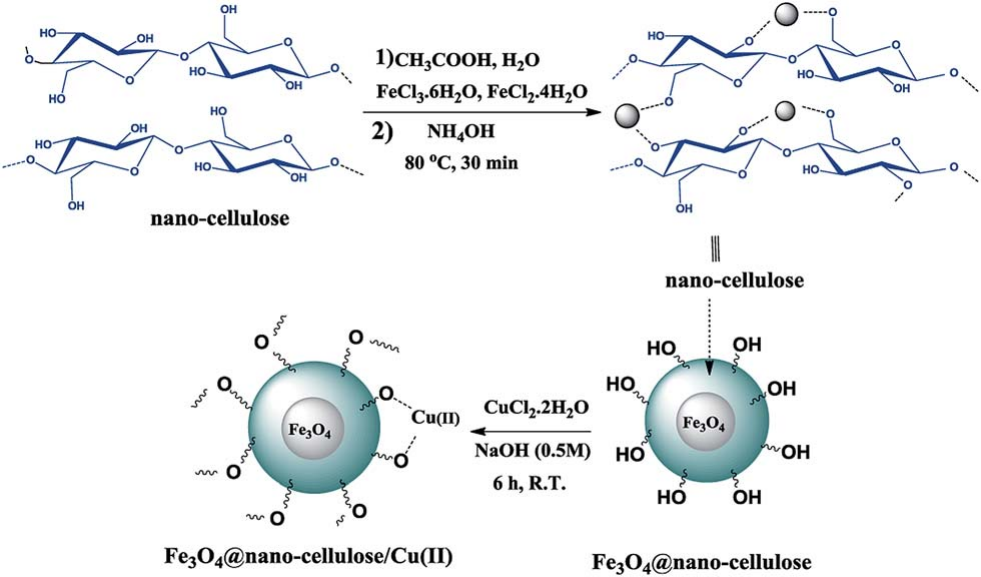
Synthesis protocol for Fe_3_O_4_@nano-cellulose/Cu(ii).

Fe_3_O_4_@nano-cellulose/Cu(ii) was prepared in a two-step process. First, Fe_3_O_4_@nano-cellulose was synthesized by co-precipitation of Fe^2+^ and Fe^3+^ ions in the presence of nano-cellulose and then it was used as a magnetic support for loading CuCl_2_ onto the cellulose section of it (Scheme 1). The magnetically heterogeneous catalyst named Fe_3_O_4_@nano-cellulose/Cu(ii), is characterized by Fourier transform infrared (FT-IR) spectroscopy, X-ray diffraction (XRD), vibrating sample magnetometer (VSM), field emission scanning electron microscopy (FESEM), transmission electron microscopy (TEM), X-ray fluorescence (XRF), energy-dispersive X-ray spectroscopy (EDS), and thermo-gravimetric analysis (TGA).

**Fig. 1:**
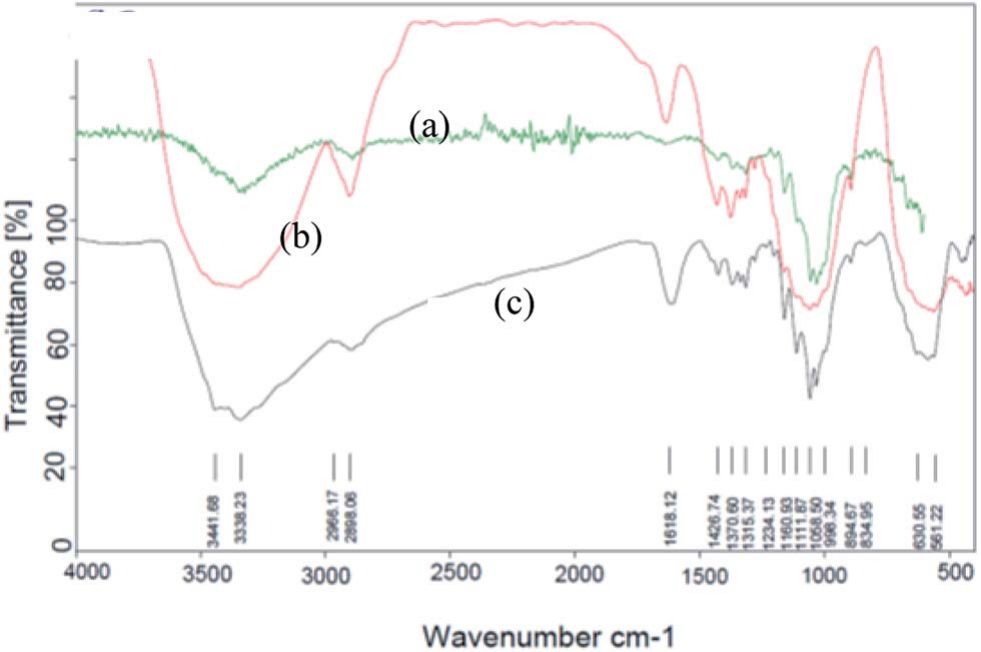
FT-IR spectra of (a) nano-cellulose, (b) Fe_3_O_4_ @ nano-cellulose and (c) Fe_3_O_4_@nano-cellulose/Cu(ii).

The FT-IR spectra of nano-cellulose, Fe_3_O_4_@nano-cellulose and Fe_3_O_4_@nano-cellulose/Cu(ii) are shown in Fig. 1.

**Fig. 2:**
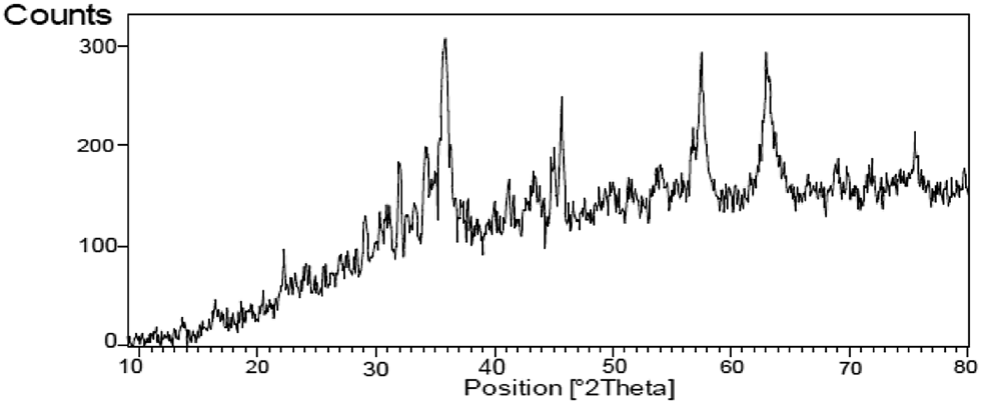
XRD pattern of Fe_3_O_4_@nano-cellulose/Cu(ii).

The FT-IR spectrum of nano-cellulose has shown a broad band at 3338 cm^−1^ which corresponds to the stretching vibrations of OH groups. The absorption bands at1058 and 1108 cm^−1^ display the stretching vibrations of the C–O bonds. For Fe_3_O_4_@nano-cellulose, in addition to the cellulose absorptions bands, stretching vibrations of Fe/O groups at 586 and 634 cm^−1^ are appeared which is indicated that the magnetic Fe_3_O_4_ nano particles are coated by nano-cellulose. The FT-IR spectrum of Fe_3_O_4_@nano-cellulose/Cu(ii) has shown a characteristic absorption band under 500 cm^−1^ that may be attributed to Cu–O band for Cu bonded to cellulose. X-ray diffraction (XRD) pattern of Fe_3_O_4_@nano-cellulose/Cu(ii) is shown in Fig. 2. Fe_3_O_4_ has shown diffraction peaks at 2*θ* = 35.79°, 43.42°, 53.94°, 57.51° and 63.08° with FWHM equal to 0.39, 0.78, 0.94, 0.31 and 0.96 respectively, which are quite matched with the cubic spinel structure of pure Fe_3_O_4_. A diffraction peaks at 2*θ* = 16.45° and 22.18° with FWHM equal to 0.23 and 0.47, respectively, has shown the existence of cellulose. Other signals in 2*θ* = 13.68, 29.10, 32.01, 34.25 and 45.71 probably reveal the existence of cellulose and bonding of Cu(ii) to cellulosic shell (Table 1).

Results of XRD analysis of Fe_3_O_4_@nano cellulose/Cu(ii)

**Table:** 

No.	1	2	3	4	5	6
Pos. [°2*θ*]	13.6815	16.4566	22.1853	29.1068	32.0094	34.2572
FWHM [°2*θ*]	0.6298	0.4723	0.2362	0.2362	0.3149	0.3149

**Table:** 

No.	7	8	9	10	11	12
Pos. [°2*θ*]	35.7935	43.4246	45.7114	53.9425	57.5101	63.0810
FWHM [°2*θ*]	0.3936	0.7872	0.3149	0.9446	0.3149	0.9600

**Fig. 3:**
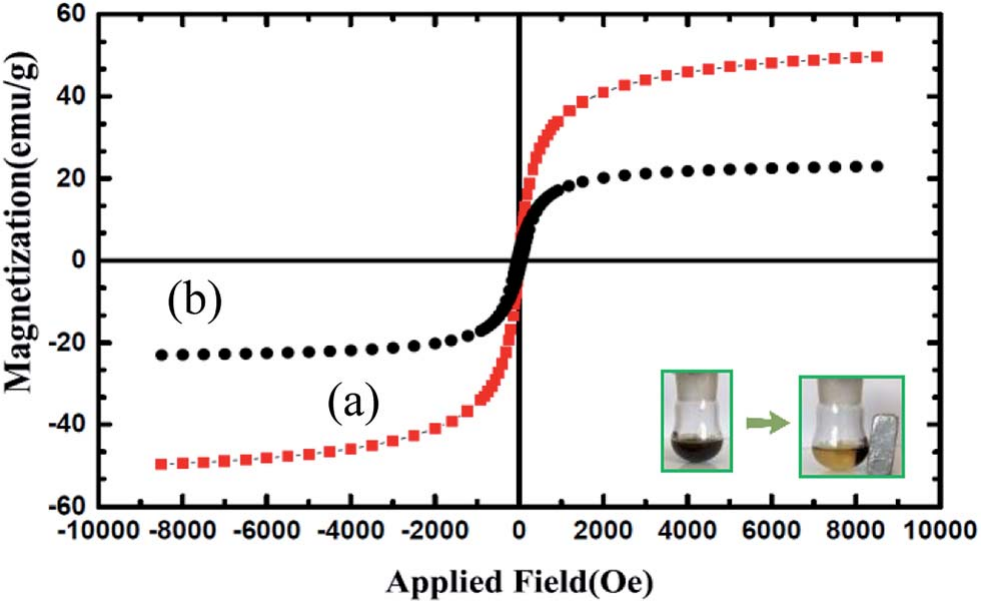
Magnetization loop of (a) Fe_3_O_4_ and (b) Fe_3_O_4_@nano-cellulose/Cu(ii).

The magnetic properties of Fe_3_O_4_ and Fe_3_O_4_@nano-cellulose/Cu(ii) were characterized at RT (300 K) by a vibrating sample magnetometer (VSM) and their hysteresis curves are presented in Fig. 3. The zero coercivity and remanence of the hysteresis loops of these magnetic nanoparticles confirm superparamagnetic property of them at room temperature. The amount of specific saturation magnetization (Ms) for Fe_3_O_4_ nanoparticles was about 50 emu g^−1^, which decreased to 25 emu g^−1^ after the bonding of Cu(ii) on the surface of Fe_3_O_4_@nano-cellulose. Despite this significant decrease, the saturated magnetization of these magnetic nanoparticles is sufficient for magnetic separation.

**Fig. 4:**
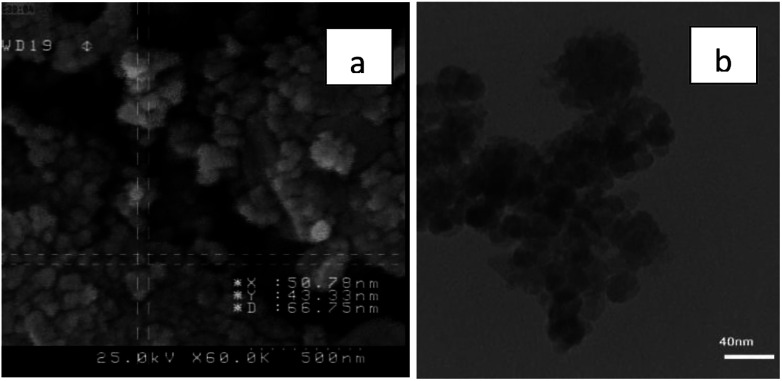
(a) FESEM image of Fe_3_O_4_@nano-cellulose/Cu(ii) and (b) TEM of Fe_3_O_4_@nano-cellulose/Cu(ii).

The particles size of Fe_3_O_4_@nano-cellulose/Cu(ii) were investigated by field emission scanning electron microscopy (FESEM) and transmission electron microscopy (TEM) in which the dimensions of them were achieved below 70 nm (Fig. 4). The chemical composition of catalyst has been measured using X-ray fluorescence (XRF) analysis (Table 2). In order to obtain the Cu : Cl ratio in Fe_3_O_4_@nano-cellulose/Cu(ii) by XRF analysis, Kilo Counts Per Seconds (KCPS) values of elements in catalyst were compared with KCPS values of the same elements in pure samples, NaCl and CuSO_4_. By this comparison, the amount of Cu and Cl were obtained 1.38 g (0.02 mol) and 0.12 g (0.003 mol), respectively. Thus, the ratio of Cu : Cl in catalyst is approximately 6 : 1.

**Table:** Results of XRF analysis of catalyst, pure NaCl and CuSO_4_

Elemental component	Fe_3_O_4_@nano-cellulose/Cu(ii)		CuSO_4_		NaCl	
	KCPS	wt%	KCPS	wt%	KCPS	wt%
CO_2_	1.5	74.8	0.2	13.3		
Fe_2_O_3_	1118.9	21.7	0.6	0.0174		
CuO	19.5	1.45	563.9	41.2		
SiO_2_	2.8	0.796	0.1	0.0403		
Na_2_O	0.9	0.573	0.2	0.218		
CaO	4.3	0.190	0.2	0.00479		
I	2.0	0.0799	1.2	0.145		
Cl	1.0	0.0681			516.5	62
Sb_2_O_3_	1.7	0.0606	0.7	0.0746		
Al_2_O_3_	0.2	0.0565				
SO_3_	0.4	0.0523	184.8	43.7		
MnO	2.2	0.0482				
MgO	0.2	0.0432	2.2	1.02		
SnO_2_	1.2	0.0340	0.7	0.0546		
Re	0.7	0.0333	0.5	0.0585		
CoO	2.2	0.0285				
Cr_2_O_3_	0.4	0.0100				
Pd	0.1	0.00860				
TiO_2_	0.3	0.00860				
Rh	0.1	0.00747				
K_2_O	0.2	0.00734				
SrO	0.5	0.00340				
Ho_2_O_3_			0.4	0.0446		
HfO_2_			1.8	0.0381		
P_2_O_5_			0.1	0.0346		
Rh			0.1	0.0213		
Total		100		100		

**Fig. 5:**
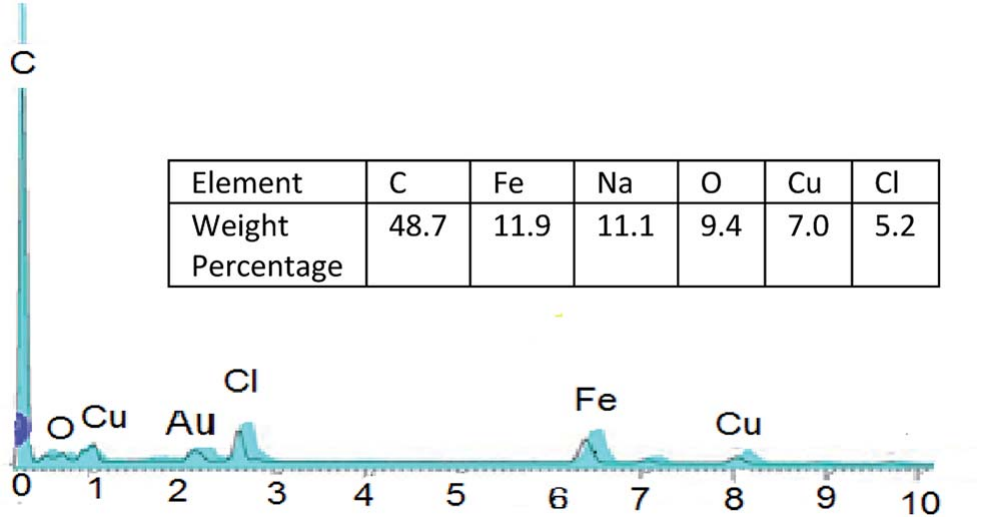
EDS (EDX) spectra of Fe_3_O_4_@nano-cellulose/Cu(ii).

And so, existence of Cu and Cl in catalyst was confirmed by EDS analysis data (Fig. 5).

**Fig. 6:**
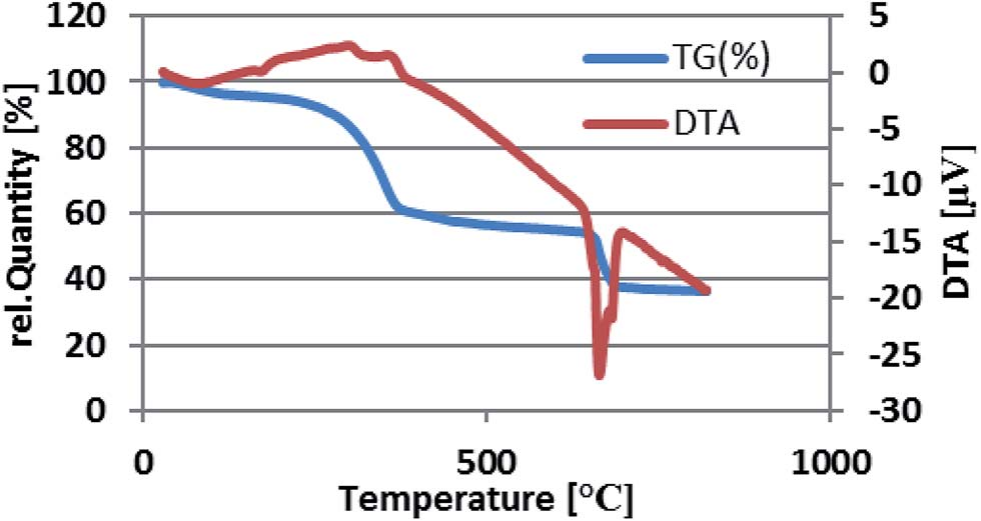
Thermal gravimetric analysis pattern of Fe_3_O_4_@nano-cellulose/Cu(ii).

The thermal stability of Fe_3_O_4_@nano-cellulose/Cu(ii) was investigated by thermo-gravimetric analysis (TGA) in the temperature range of 30–800 °C (Fig. 6).

The TGA curve illustrates four mass-loss steps. Firstly, a very small weight loss (2.53%) from 50 to 100 °C is corresponded to remove of catalyst moisture. Subsequently, the main weight loss step in the temperature ranges 200–370 °C (33%) is attributed to the decomposition of cellulose units through the formation of levoglucosan and other volatile compounds. Finally, there are two weight loss steps in the temperature ranges 400–600 and 650–690 °C (5 and 16%, respectively). According to the TG–DTA diagram of Fe_3_O_4_@nano-cellulose/Cu(ii), it was revealed that this catalyst is suitable for the promotion of organic reactions below 200 °C.

### Catalyst efficiency for synthesis of 4*H*-pyrimido[2,1-*b*]benzothiazole derivatives

After characterization of Fe_3_O_4_@nano-cellulose/Cu(ii), the activity of catalyst was evaluated for the synthesis of 4*H*-pyrimido[2,1-*b*]benzothiazole derivatives.

For optimization of the reaction conditions, the reaction of 2-aminobenzothiazole, 4-nitrobenzaldehyde and ethyl acetoacetate as a model reaction was investigated (Table 3). As shown in Table 3, entry 14, it was found that 0.03 g of Fe_3_O_4_@nano-cellulose/Cu(ii) under solvent-free condition at 80 °C is the best reaction condition. In order to compare the efficiency of present nano-catalyst with other catalysts, the model reaction was also performed using the reported catalysts for the synthesis of 4*H*-pyrimido[2,1-*b*]benzothiazole derivatives. As Table 4 indicates, in comparison with other reported catalysts, we have found that Fe_3_O_4_@nano-cellulose/Cu(ii) promoted reaction has shorter reaction time, higher yields of products, green reaction conditions and simpler workup. Finally, the above optimized reaction conditions were explored for the synthesis of 4*H*-pyrimido[2,1-*b*]benzothiazole derivatives and the results are summarized in Table 5. The reusability of the catalyst was also investigated on the model reaction. The magnetic nature of the catalyst allowed its facile recovery by simple separation by an external magnet, washing with ethanol and drying at room temperature to provide an opportunity for recycling experiments. The separated nano-catalyst was reused in the above-mentioned reaction for the synthesis of IV_b_ for four times without considerable loss of its catalytic activity (Table 3). Partial loss of activity may be due to blockage of catalyst active sites and/or partial leaching of Cu from the catalyst.

**Table:** The reaction of 2-aminobenzothiazole, 4-nitrobenzaldehyde, and ethyl acetoacetate in the presence of Fe_3_O_4_@nano-cellulose/Cu(ii) under various conditions^a^

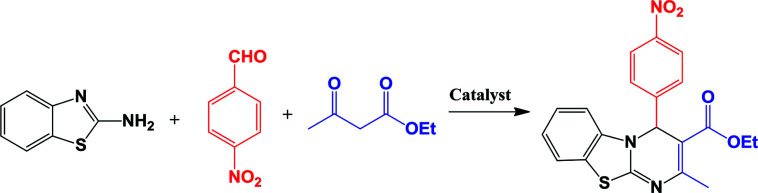					
Entry	Solvent	Catalyst (g)	Condition	Time (h)	Yield^b^ (%)
1	—	—	80 °C	7 h	30
2	—	CuCl_2_	80 °C	3h	69
3	—	Fe_3_O_4_	70 °C	3h	37
4	—	Fe_3_O_4_@nano-cellulose	80 °C	4 h	41
5	C_2_H_5_OH	—	R. T	7 h	—
6	C_2_H_5_OH	Catalyst (0.04)^c^	R. T	3 h	35
7	C_2_H_5_OH	Catalyst (0.04)^c^	Reflux	3 h	57
8	H_2_O	Catalyst (0.04)^c^	Reflux	3 h	42
9	CH_3_OH	Catalyst (0.04)^c^	Reflux	3 h	51
10	—	Catalyst (0.04)^c^	R. T	3 h	43
11	—	Catalyst (0.04)^c^	70 °C	1 h	85
12	—	Catalyst (0.05)^c^	80 °C	0.5	93
13	—	Catalyst (0.04)^c^	80 °C	0.5	97
14	—	Catalyst (0.03)^c^	80 °C	0.5	97
15	—	Catalyst (0.02)^c^	80 °C	0.5	84
16	—	Catalyst (0.03), 2^th^run^c^	80 °C	0.5	93
17	—	Catalyst (0.03), 3^rd^run^c^	80 °C	0.5	88
18	—	Catalyst (0.03), 4^th^run^c^	80 °C	0.5	83

a The amount ratio of 2-aminobenzothiazole (mmol), 4-nitrobenzaldehyde (mmol) and ethyl acetoacetate (mmol) are equal to 1 : 1 : 1.

b Isolated yield.

c Fe_3_O_4_@ nano-cellulose/Cu(ii).

**Table:** Comparative study of the present method and some other reported methods for synthesis of 4*H*-pyrimido[2,1-*b*]benzothiazole derivatives

Ent.	Solvent	Catal.	Tem. (°C)	Time (h)	Yield^a^ (%)	Ref.
1	CH_3_OH	Acetic acid (20 mol%)	65	18	62	20
2	EG	TBAHS (30 mol%)^b^	120	2	72	17
3	HOAc	Chitosan (0.080 g)	70	1.6	93	18
4	—	TMGT (0.080 g)^c^	100	5	53	16
5	—	AlCl_3_ (10 mol%)	65	1.2	97	22
6	—	Fe_3_O_4_@NCs/TiCl (0.03 g)	70	0.6	96	23
7	—	Fe_3_O_4_@ nano-cellulose/Cu(ii) (0.03 g)	80	0.5	97	This work

a Isolated yield.

b Tetrabutylammonium hydrogen sulfate.

c 1,1,3,3-*N*,*N*,*N*′,*N*′-Tetramethylguanidinium trifluoroacetate.

**Table:** Synthesis of 4*H*-pyrimido[2,1-*b*]benzothiazole derivatives (IV_a–m_) in the presence of Fe_3_O_4_@ nano-cellulose/Cu(ii) under solvent-free condition at 80 °C^a^ appear here with headings as appropriate

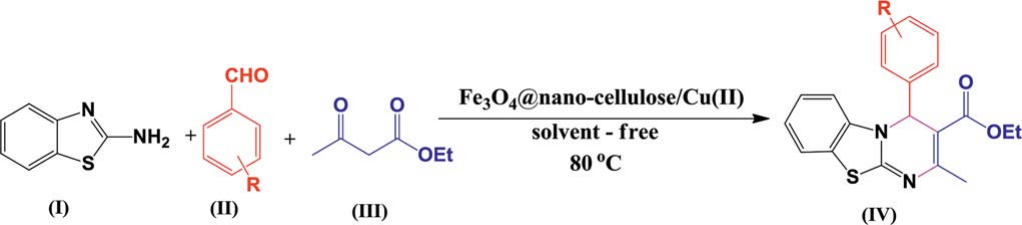							
Ent.	R	Prod.	Time (min)	Yield^b^ (%)	M. P.		Ref.
					Found	Report	
1	H–	IV_a_	45	84	178–180	177–179	17
2	4-NO_2_–	IV_b_	30	97	171–173	170–172	22
3	4-Cl–	IV_c_	30	95	87–89	86–88	21
4	4-Br–	IV_d_	30	97	110–114	110–114	16
5	4-OH–	IV_e_	60	82	210–212	210–212	22
6	2-NO_2_–	IV_f_	45	88	122–125	122–125	23
7	2-Cl–	IV_g_	40	87	124–126	125–127	17
8	2-EtO–	IV_h_	60	75	171–175	171–175	23
9	3-NO_2_–	IV_i_	35	93	222–224	222–224	21
10	3-OH–	IV_j_	65	79	260–263	260–263	23
11	2,4-(Cl)_2_–	IV_k_	45	85	133–135	133–135	17
12	2,4-(MeO)_2_–	IV_l_	75	74	164–166	164–166	23
13	3,4-(OH)_2_–	IV_m_	70	71	225–227	225–227	23

a I (mmol) : II (mmol) : III (mmol) : Fe_3_O_4_@ nano-cellulose/Cu(ii) (g) is equal to 1 : 1 : 1 : 0.03.

b Isolated yield.

**Scheme 2:**
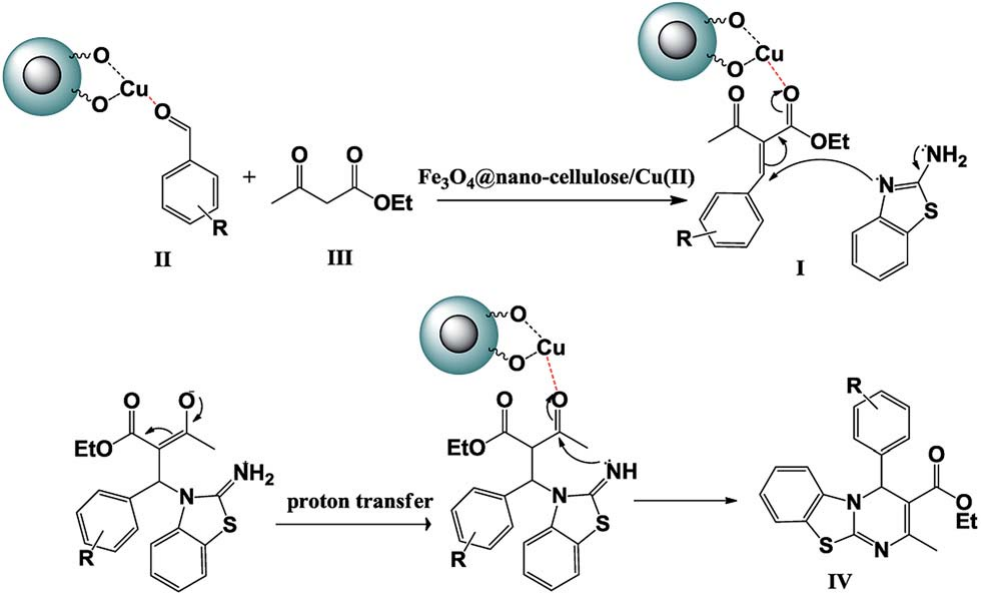
Proposed mechanism for the synthesis of 4*H*-pyrimido [2,1-*b*]benzothiazole derivatives IV_a–m_.

Substituents on the aldehyde showed a significant effect in terms of the yield and reaction time under the optimized reaction conditions. The electron-withdrawing groups increase rate and yields of reaction compared to electron-donating groups. Suggested mechanism for the synthesis of 4*H*-pyrimido[2,1-*b*]benzothiazole (IV) in presence of Fe_3_O_4_@ nano-cellulose/Cu(ii) was shown in Scheme 2. Cu(ii) activate the carbonyl group of benzaldehyde (II) for Knoevenagel reaction with β-ketoesters (III) to production of intermediate (I). Meanwhile, Cu(ii) activate the carbonyl group in intermediate (I) for Michael addition with 2-aminobenzothiazole and then interamolecular cyclization to production of product (IV).

The structures of the products IV_a–m_ were studied by their melting point, IR and ^1^H NMR spectra. In the FTIR spectra of products, the ester C=O stretching vibration band is appeared at 1690 cm^−1^ due to conjugation.

## Conclusions

We have demonstrated the preparation and characterization of Fe_3_O_4_@ nano-cellulose/Cu(ii) as a novel magnetite recoverable, eco-friendly, inexpensive and efficient nanocatalyst. The catalytic activity of the prepared catalyst was investigated in the synthesis of 4*H*-pyrimido[2,1-*b*] benzothiazole derivatives through one-pot three-component reaction of aldehydes, ethyl acetoacetate, and 2-aminobenzothiazole under solvent-free condition at 80 °C. This protocol includes some important advantages such as mild reaction conditions, short reaction time, excellent yields, easy work-up, high purity of products. And so, magnetic separation and reusability of nanocatalyst is other advantages of this protocol.

## Experimental

### General remarks

All compounds were purchased from Aldrich, Merck, and Fluka chemical companies. Nano-cellulose and Fe_3_O_4_@nano-cellulose were synthesized *via* our previously reported methods.^23^ FT-IR spectra were run on a Bruker, Equinox 55 spectrometer. A Bruker (DRX-400 Avance) NMR was used to record the ^1^H NMR and ^13^C NMR spectra. The X-ray diffraction (XRD) pattern was obtained by a Philips Xpert MPD diffractometer equipped with a Cu Kα anode (*k* = 1.54 A°) in the 2*θ* range from 10 to 80°. XRF analysis was done with Bruker, S4 Explorer instrument. VSM measurements were performed by using a vibrating sample magnetometer (Meghnatis Daghigh Kavir Co. Kashan, Iran). Melting points were determined by a Buchi melting point B-540 B.V.CHI apparatus. Field emission scanning electron microscopy (FESEM) image was obtained on a Mira 3-XMU. Transmission electron microscopy (TEM) image was obtained using a Philips CM120 with a LaB6 cathode and accelerating voltage of 120 kV. energy-dispersive X-ray spectroscopy (EDS) of Fe_3_O_4_@nano-cellulose/Cu(ii) was measured by an EDS instrument and Phenom pro X. Thermal gravimetric analysis (TGA) was conducted using “STA 504” instrument.

### Preparation of Fe_3_O_4_@nano-cellulose/Cu(ii)

In a flask containing 50 ml of 0.5 M NaOH, Fe_3_O_4_@nano-cellulose (0.5 g) was added with stirring. Then, 75 ml of CuCl_2_ aqueous solution, 0.04 M, was added. A dark blue solution was obtained immediately that was stirred at room temperature. After 6 h, the magnetically heterogeneous catalyst, Fe_3_O_4_@nano-cellulose/Cu(ii), removed from solution by an external magnet. The catalyst washed with ethanol and water two times and dried at an oven at 80 °C.

### General procedure for synthesis of 4*H*-pyrimido[2,1-*b*]benzothiazole derivatives

A mixture of 2-aminobenzothiazole (1 mmol), aldehyde (1 mmol), ethyl acetoacetate (1 mmol) and Fe_3_O_4_@nano-cellulose/Cu(ii) (0.03 g) was heated at 80 °C. After completion of the reaction (monitored by TLC), the reaction mixture was dissolved in hot ethanol (3 ml) and the catalyst was separated by using an external magnet. Subsequently by adding water to the decanted solution, the product was appeared as a pure solid in high yields. The recovered catalyst was washed 3 times with ethanol, dried and reused for subsequent runs under the same reaction conditions.

## Conflicts of interest

There are no conflicts to declare.

## Supplementary Material

RA-009-C8RA09203F-s001
