# Supplementary material for: Fe3O4@nano-cellulose/Cu(ii): a bio-based and magnetically recoverable nano-catalyst for the synthesis of 4H-pyrimido[2,1-b]benzothiazole derivatives
Source: RSC Adv. 2019 Jan 11;9(3):1278–83. doi: 10.1039/c8ra09203f (PMC9059560; doi:10.1039/c8ra09203f)
Supplement: RA-009-C8RA09203F-s001 [file RA-009-C8RA09203F-s001.pdf]

Electronic Supplementary Material (ESI) for RSC Advances.
This journal is © The Royal Society of Chemistry 2018

Fe_3O_4@nano-cellulose/Cu (II): a bio-based and magnetically recoverable nano-catalyst for the synthesis of 4*H*-pyrimido[2,1-*b*]benzothiazole derivatives

Nasrin safajoo[1], Bi Bi Fatemah Mirjalili[1,*], Abdolhamid Bamoniri[2]

[1]Department of Chemistry, College of Science, Yazd University, Yazd, P.O.Box 89195-741, Iran, E-mail:fmirjalili@yazd.ac.ir, Telephone: +983531232672, Fax: +98 3538210644
[2]Department of Organic Chemistry, Faculty of Chemistry, University of Kashan, Kashan, I.R.IRAN.

Ethyl-2-methyl-4-(phenyl)-4*H*-pyrimido[2,1-*b*][1,3]benzothiazole-3-carboxylate (table 5, IV$_a$).

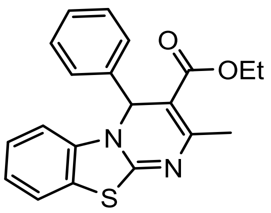

Pale yellow solid. ^{1}H NMR (Acetone-d$_6$, 400 MHz): δ 7.70 (dd, *J*=8, 0.8 Hz, 1H), 7.52-7.54 (m, 2H), 7.40 (dd, *J*=8, 1.2 Hz, 1H), 7.30-7.35 (m, 3H), 7.19-7.26 (m, 2H), 6.54 (s, 1H), 4.07-4.20 (m, 2H), 2.38 (s, 3H), 1.27 (t, 3H, J=7.2 Hz). IR (KBr): 2974, 1669, 1594, 1460, 1242, 747 cm^{-1}. mp: 178-180 ˚C.

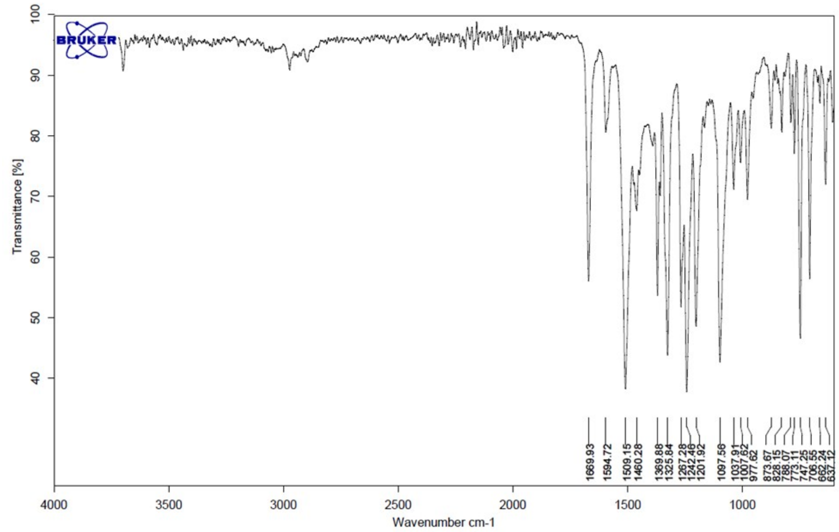

The FT-IR spectrum of product (IV$_a$)

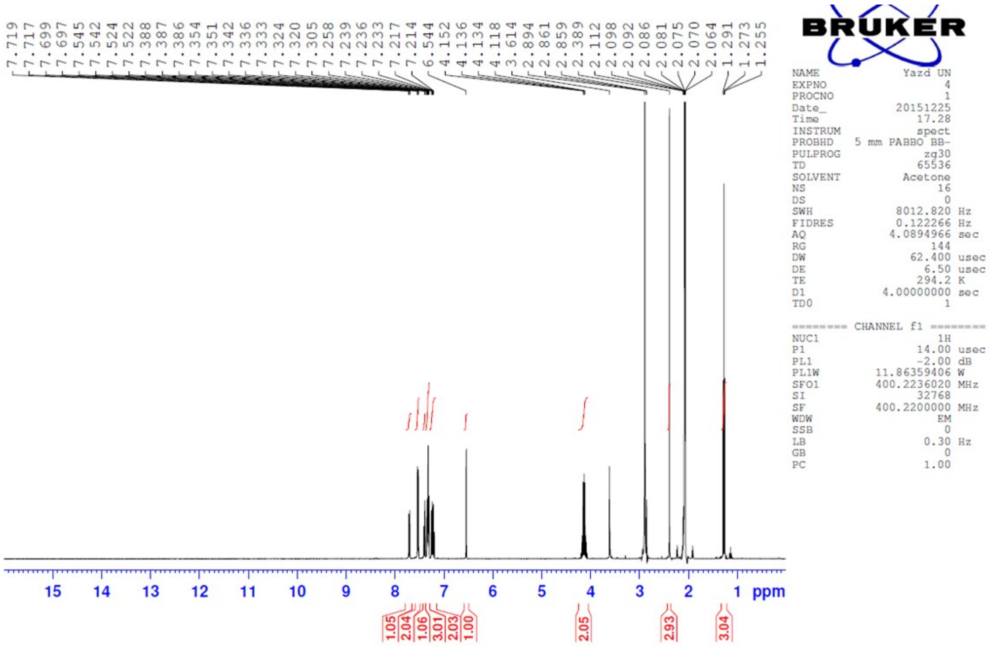

The ^{1}H NMR (400MHz) spectrum of product (IV_a)

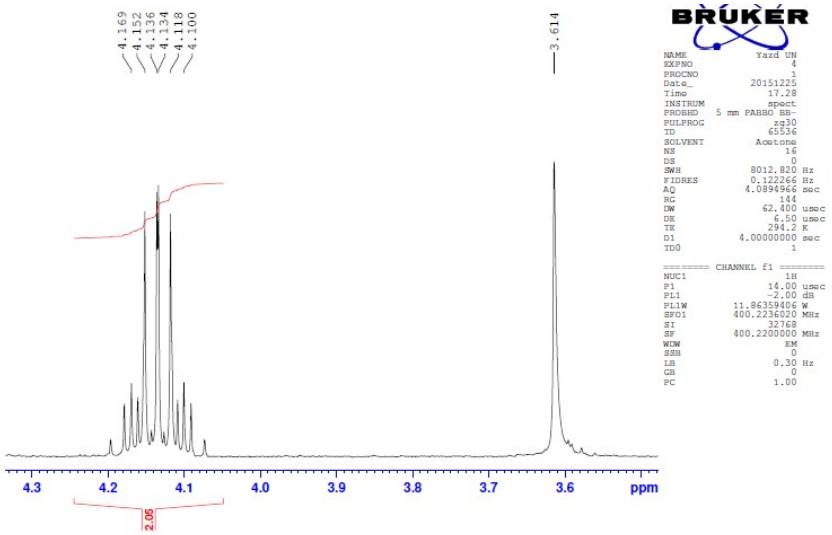

The ^{1}H NMR (400MHz) spectrum of product (IV_a)

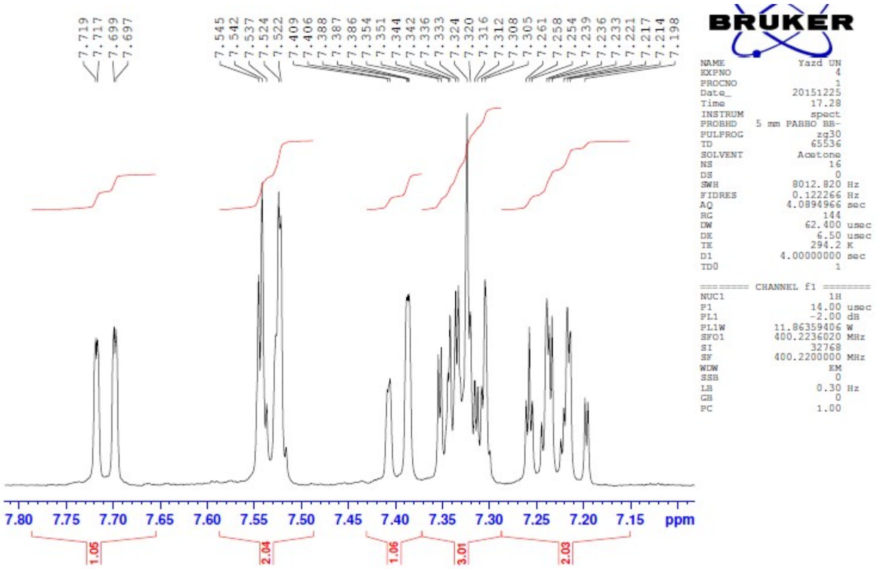

The ^{1}H NMR (400MHz) spectrum of product (IV_a)

Ethyl-2-methyl-4-(4-nitrophenyl)-4*H*-pyrimido[2,1-*b*][1,3]benzothiazole-3-carboxylate

(table 5, IV_b).

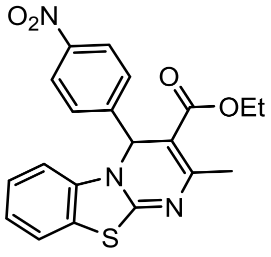

Yellow solid. ^{1}H NMR (Acetone-d_6, 400 MHz): δ 8.18 (d, *J*=8.8 Hz, 2H), 7.80 (d, *J*=8 Hz, 2H), 7.72 (d, *J*=8 Hz, 1H), 7.41 (d, *J*=8 Hz, 1H), 7.32 (t, *J*=8 Hz, 1H), 7.22 (t, *J*=7.2 Hz, 1H), 6.69 (s, 1H), 4.09-4.16 (m, 2H), 2.37 (s, 3H), 1.25 (t, *J*=7.2 Hz, 3H). IR (KBr): 3074, 2981, 1700, 1670, 1585, 1501, 1347, 1272, 1242, 1202, 745 cm^{-1}. mp: 171-173 ˚C.

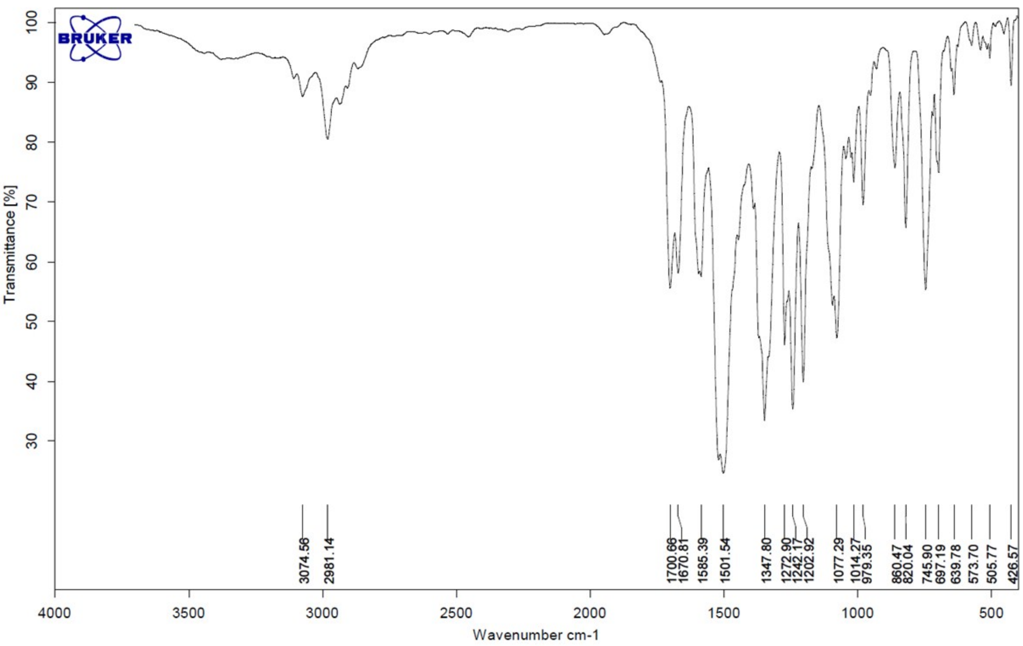

The FT-IR spectrum of product (IV_b)

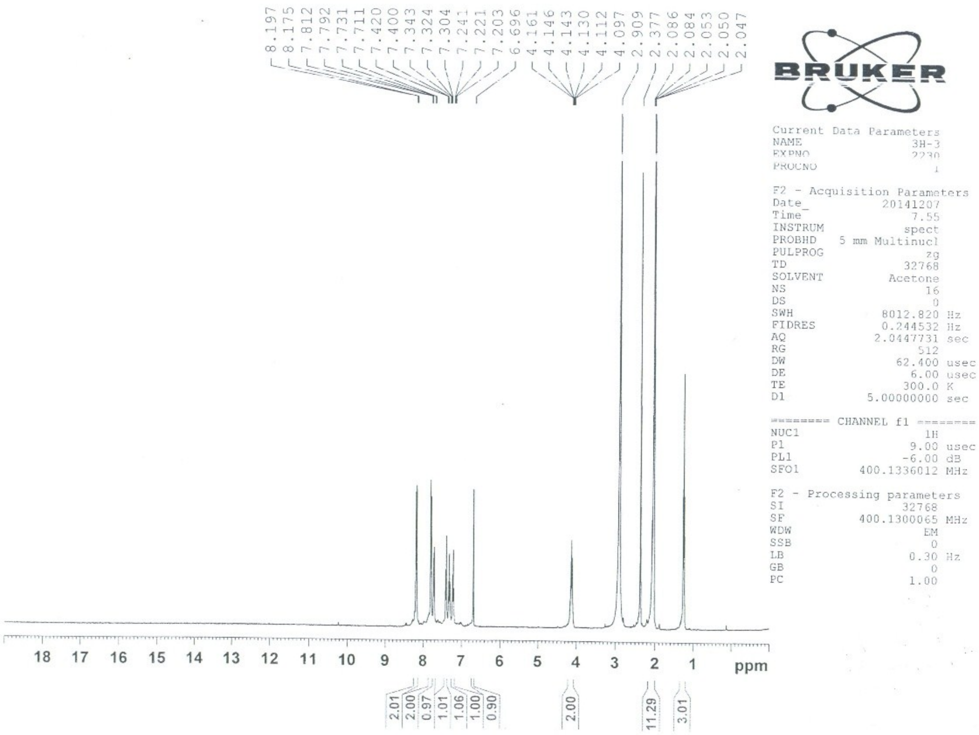

The ^{1}H NMR (400MHz) spectrum of product (IV_b)

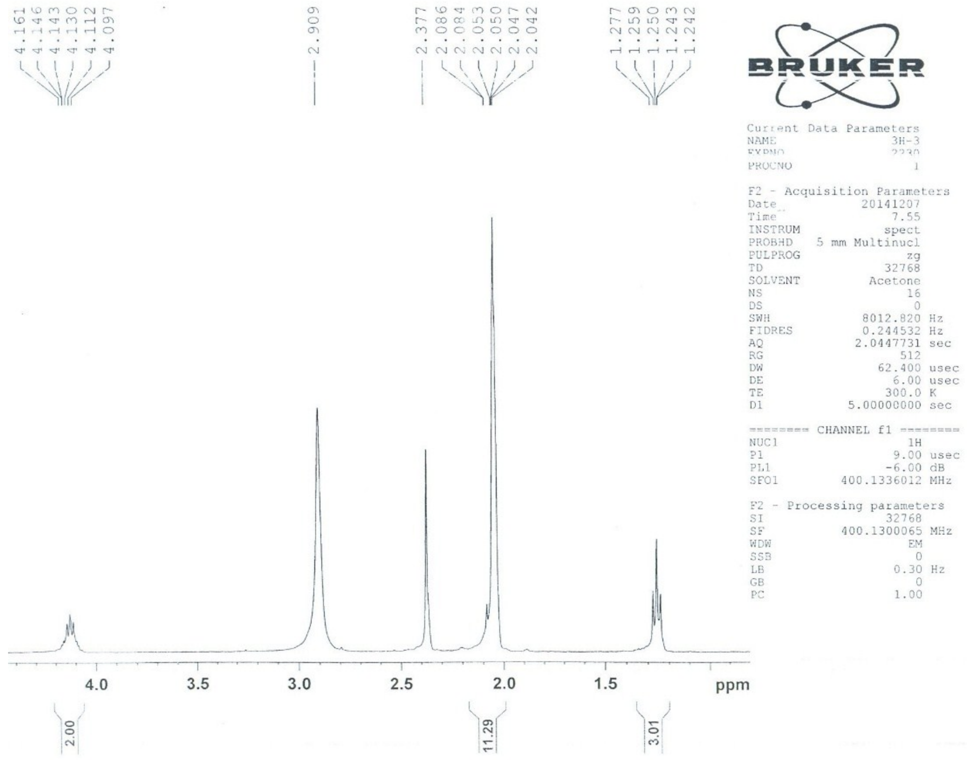

The ^{1}H NMR (400MHz) spectrum of product (IV_b)

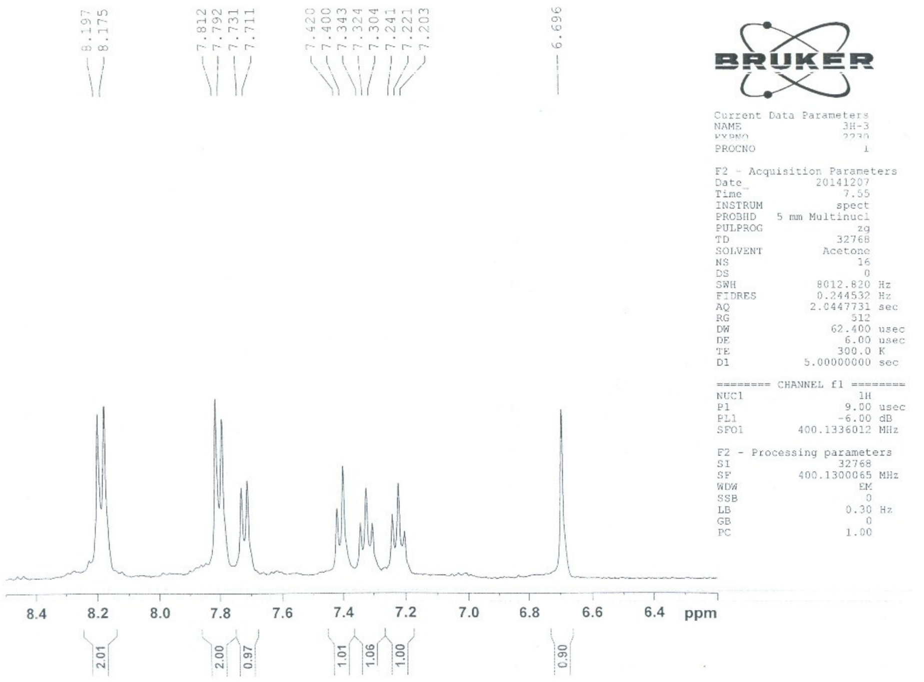

The ^{1}H NMR (400MHz) spectrum of product (IV_b)

Ethyl-2-methyl-4-(4-chlorophenyl)-4*H*-pyrimido[2,1-*b*][1,3]benzothiazole-3-carboxylate (table 5, IV_c).

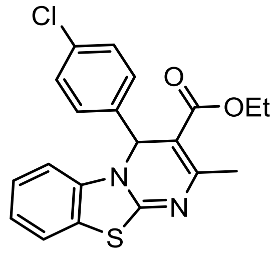

Yellow solid. ^{1}H NMR (Acetone-d_6, 400 MHz): δ 7.70 (d, *J*=8 Hz, 1H), 7.50-7.55 (m, 2H), 7.30-7.41 (m, 4H), 7.21 (td, *J*=7.2, 1.2 Hz, 1H), 6.54 (s, 1H), 4.05-4.12 (m, 2H), 2.36 (s, 3H), 1.20-1.30 (m, 3H). IR (KBr): 2978, 1687, 1580, 1488, 1239, 1200, 1074, 833, 743 cm^{-1}. mp: 87-89 ˚C.

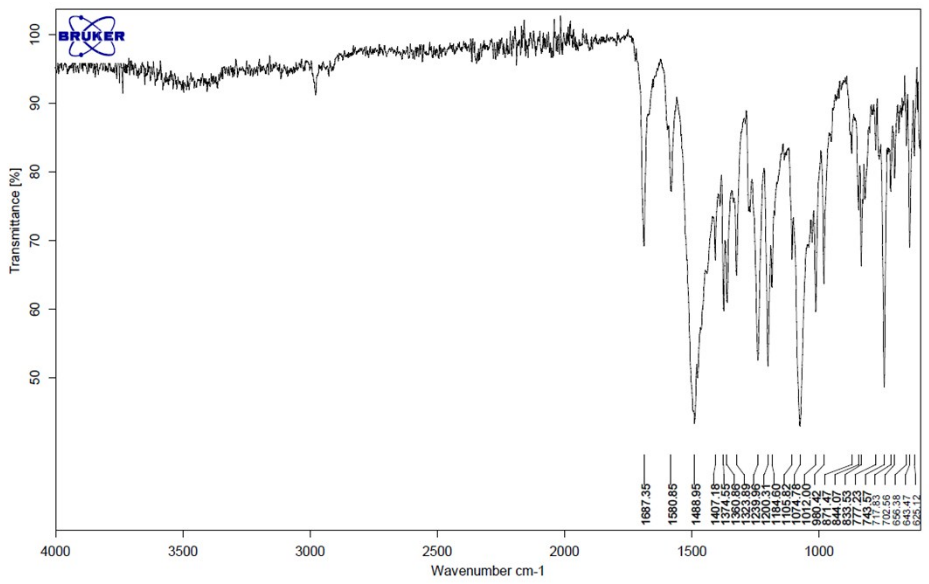

The FT-IR spectrum of product (IV_c)

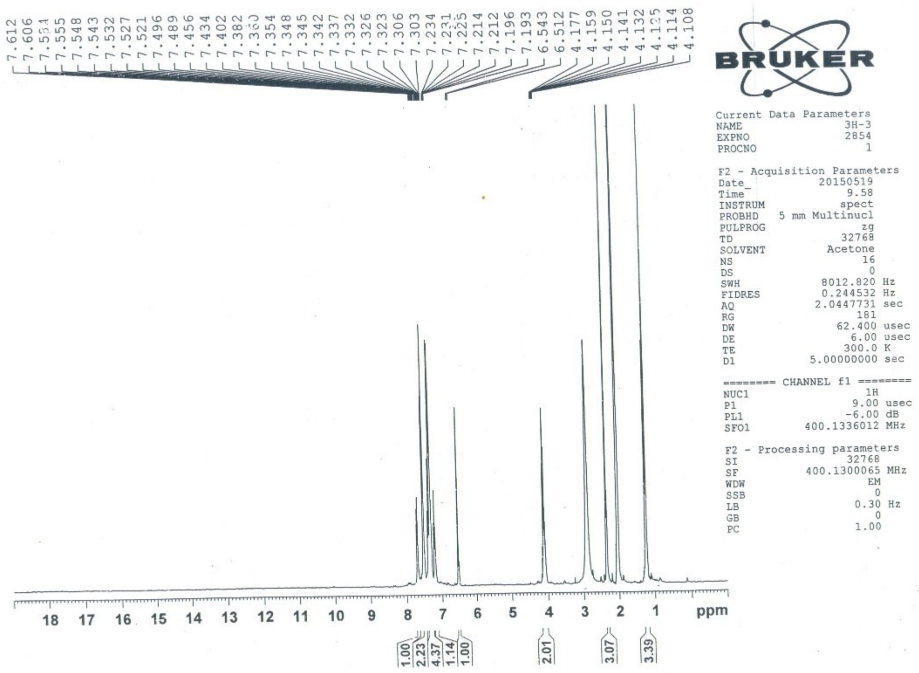

The ^{1}H NMR (400MHz) spectrum of product (IV_c)

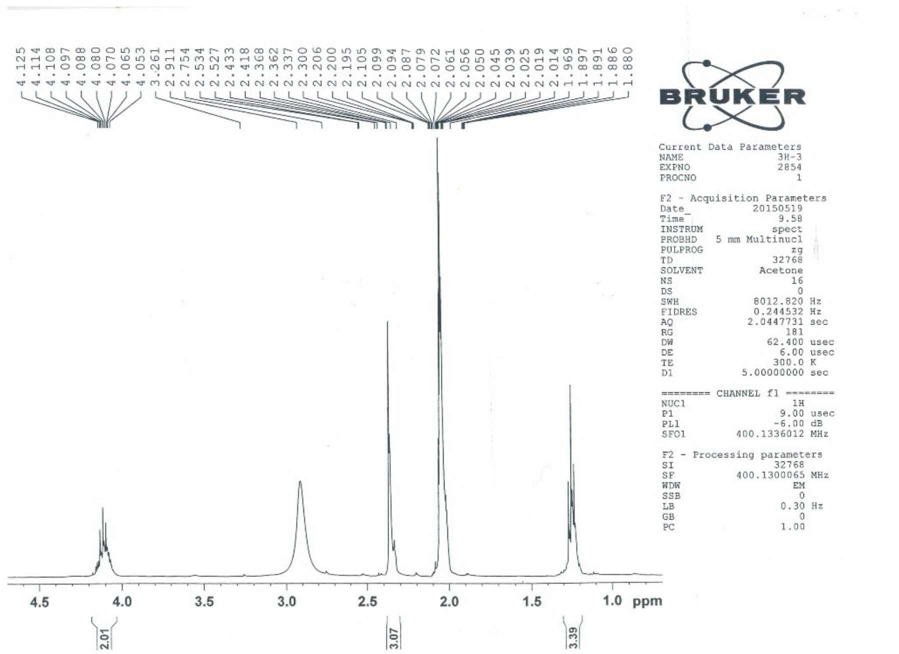

The ^{1}H NMR (400MHz) spectrum of product (IV_c)

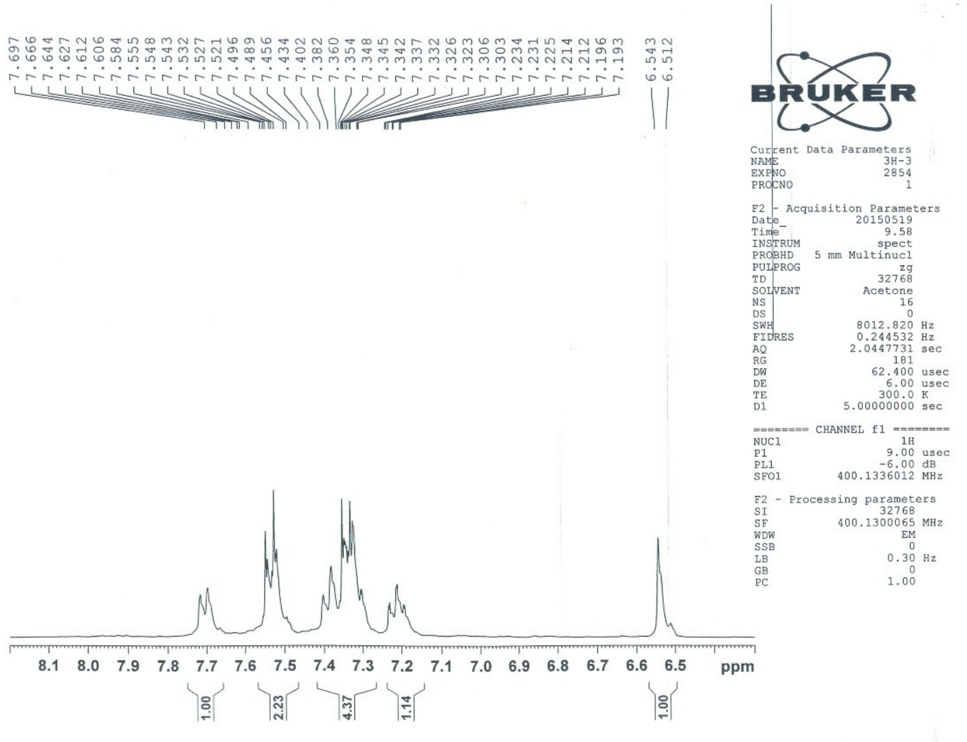

The ^{1}H NMR (400MHz) spectrum of product (IV_c)

Ethyl-2-methyl-4-(4-bromo phenyl)-4*H*-pyrimido[2,1-*b*][1,3]benzothiazole-3-carboxylate (table 5, IV_d).

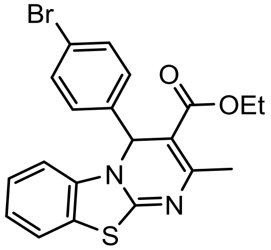

Orange solid. ^{1}H NMR (Acetone-d_6, 400 MHz): δ 7.70 (d, *J=8* Hz, 1H), 7.43-7.51 (m, 4H), 7.38 (d, *J*=8 Hz, *1H*), 7.32 (td, *J*=8, *1.2, Hz,* 1H), 7.20 (td, *J*=8, *1.2, Hz, 1H*), 6.52 (s, 1H), 4.07-4.15 (m, 2H), 2.37 (s, 3H), 1.20 (t, 3H). IR (KBr): 1693, 1582, 1482, 1406, 1270, 1239, 1201, 1069, 873, 743 cm^{-1}. mp: 110-114 ˚C.

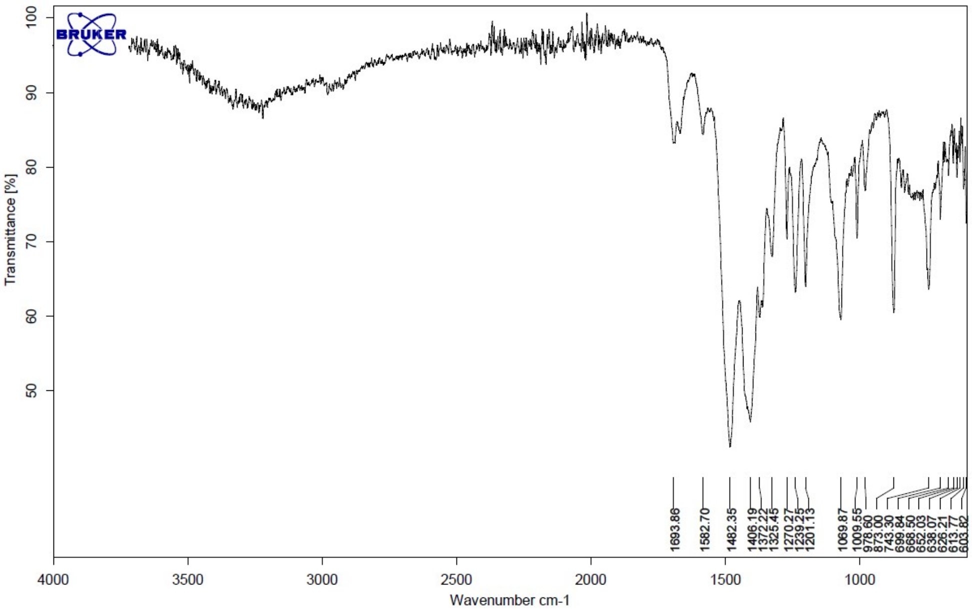

The FT-IR spectrum of product (IV_d)

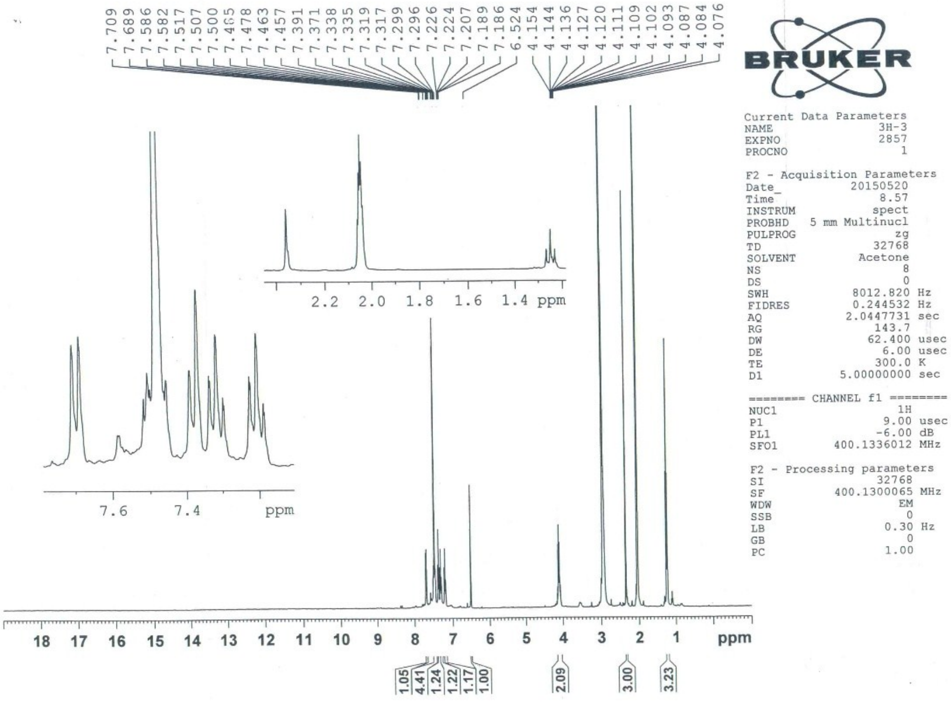

The ^{1}H NMR (400MHz) spectrum of product (IV_d)

Ethyl-2-methyl-4-(4-hydroxy phenyl)-4*H*-pyrimido[2,1-*b*][1,3]benzothiazole-3-carboxylate (table 5, IV$_e$).

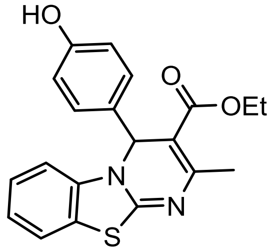

Pale yellow solid. ^{1}H NMR (Acetone-d$_6$, 400 MHz): δ 8.10-8.20 (m, 1H), 7.80-7.91 (m, 1H), 7.40-7.60 (m, 5H), 6.80-690 (m, 2H), 6.78 (s, 1H), 4.10-4.40 (m, 2H), 2.60 (s, 3H), 1.30 (m, 3H). IR (KBr): 3402, 2887, 1718, 1611, 1540, 1514, 1465, 1343, 1277, 1242, 1113, 963, 843 cm^{-1}. mp: 210-212 ˚C.

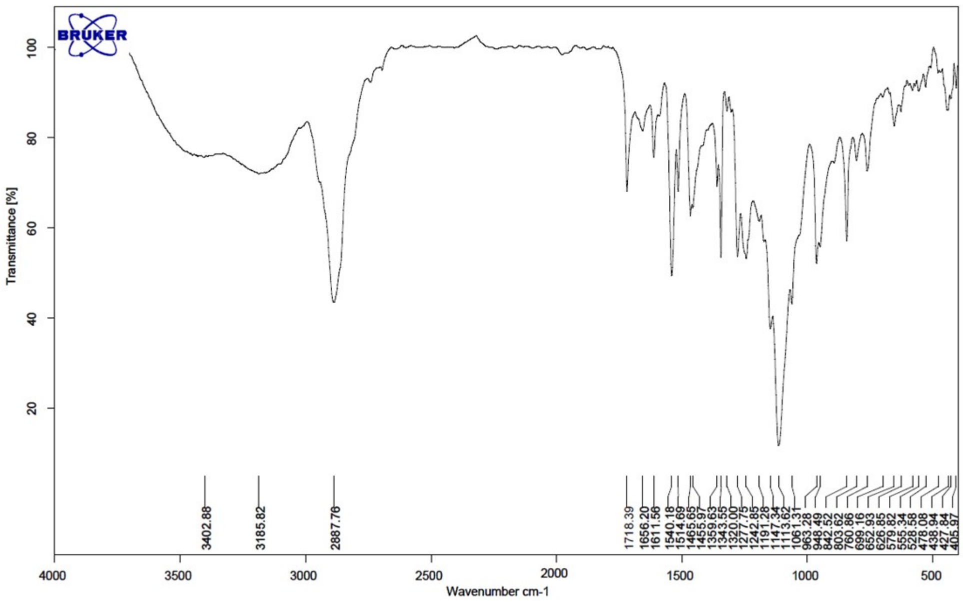

The FT-IR spectrum of product (IV$_e$)

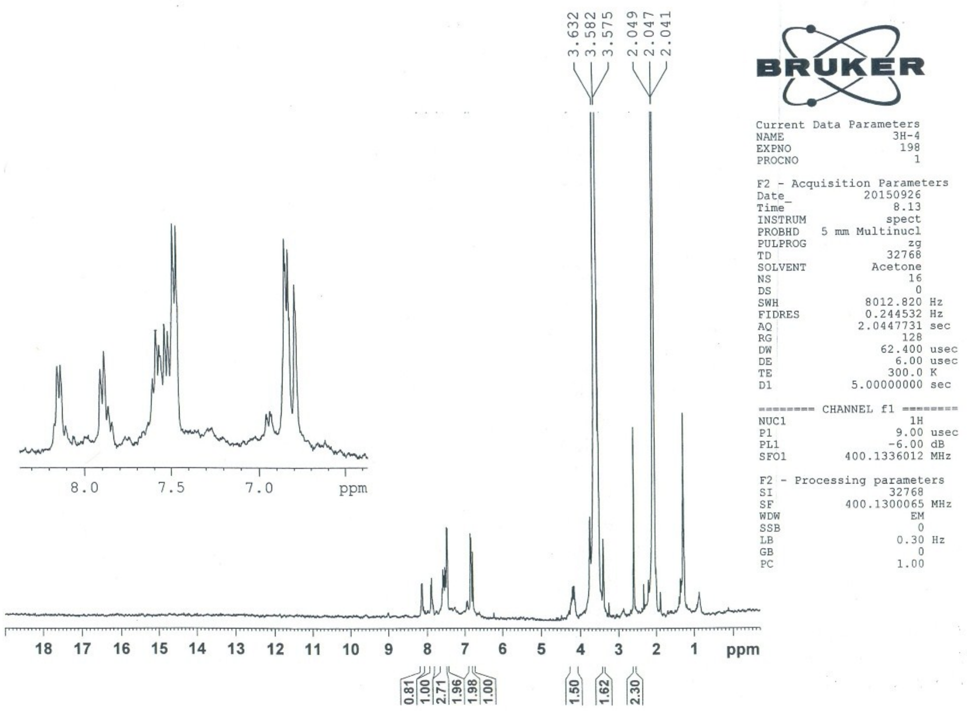

The ^{1}H NMR (400MHz) spectrum of product (IV_e)

Ethyl-2-methyl-4-(2-nitrophenyl)-4*H*-pyrimido[2,1-*b*][1,3]benzothiazole-3-carboxylate

(table 5, IV_f).

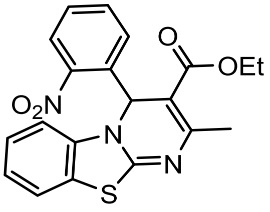

Red orange solid. ^{1}H NMR (Acetone-d_6, 400 MHz): δ 7.70 (m, 2H), 7.49 (d, *J*=8 Hz, 1H), 7.30-7.38 (m, 3H), 7.27 (td, *J*=6.4, 1.2 Hz, 1H), 7.22 (t, *J*=7.6 Hz, 1H), 6.82 (s, 1H), 4.11 (q, *J*=7.2 Hz, 2H), 2.37 (s, 3H), 1.20 (t, J=7.2 Hz, 3H). IR (KBr): 2981, 1667, 1585, 1504, 1443, 1361, 1329, 1241, 1201, 1097, 743 cm^{-1}. mp: 122-125 °C.

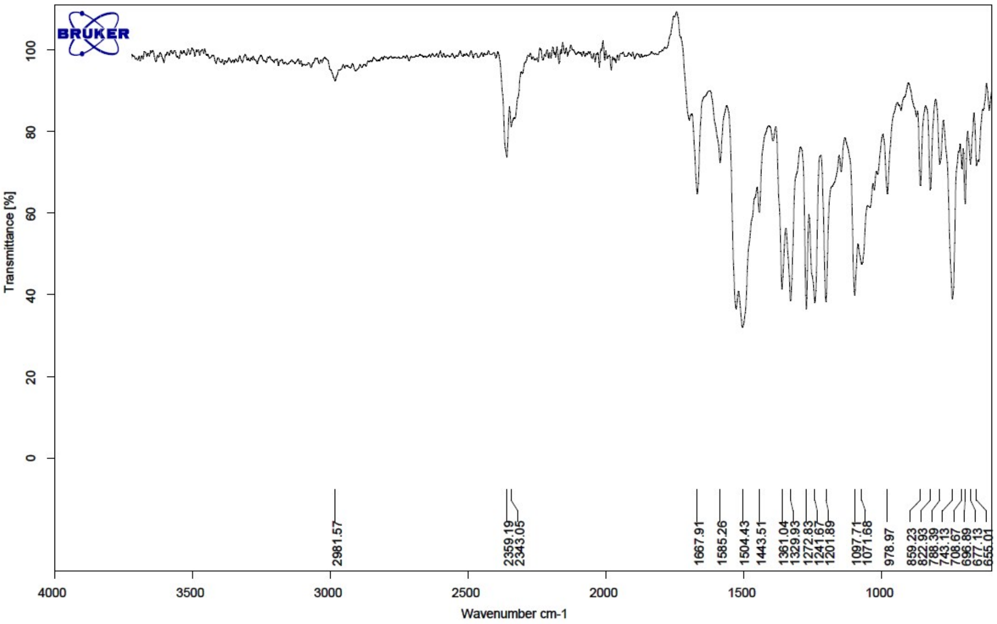

The FT-IR spectrum of product (IV_f)

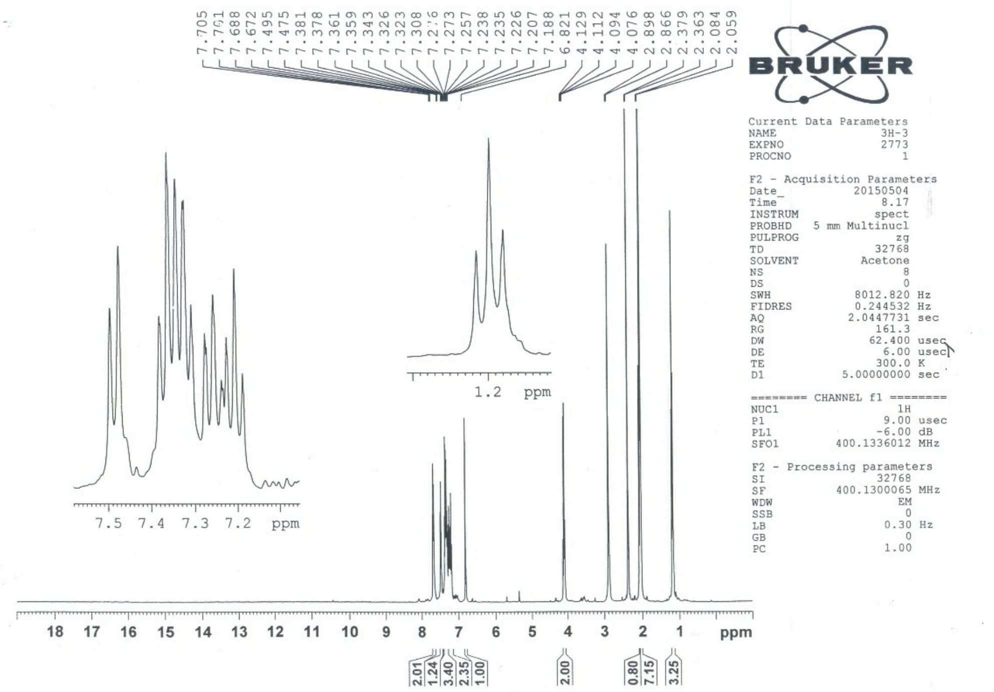

The ^{1}H NMR (400MHz) spectrum of product (IV_f)

Ethyl-2-methyl-4-(2-chlorophenyl)-4*H*-pyrimido[2,1-*b*][1,3]benzothiazole-3-carboxylate

(table 5, IV_g).

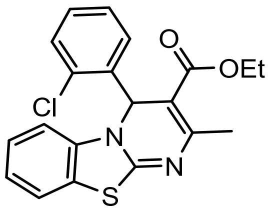

Yellow solid. ^{1}H NMR (Acetone-d_6, 400 MHz): δ 7.94 (d, J=8 Hz, 1H), 7.84 (d, J=8 Hz, 1H), 7.74 (d, J=8 Hz, 1H), 7.67 (m, 1H), 7.49-7.54 (m, 2H), 7.38 (t, J=7.2 Hz, 1H), 7.25-7.31 (m, 2H), 3.97-3.99 (m, 1H), 4.01-4.15 (m, 1H), 2.30 (s, 1H), 1.2 (t, 3H). IR (KBr): 2969, 1675, 1593, 1473, 1367, 1325, 1268, 1237, 1096, 746 cm^{-1}. mp: 124-126 ˚C.

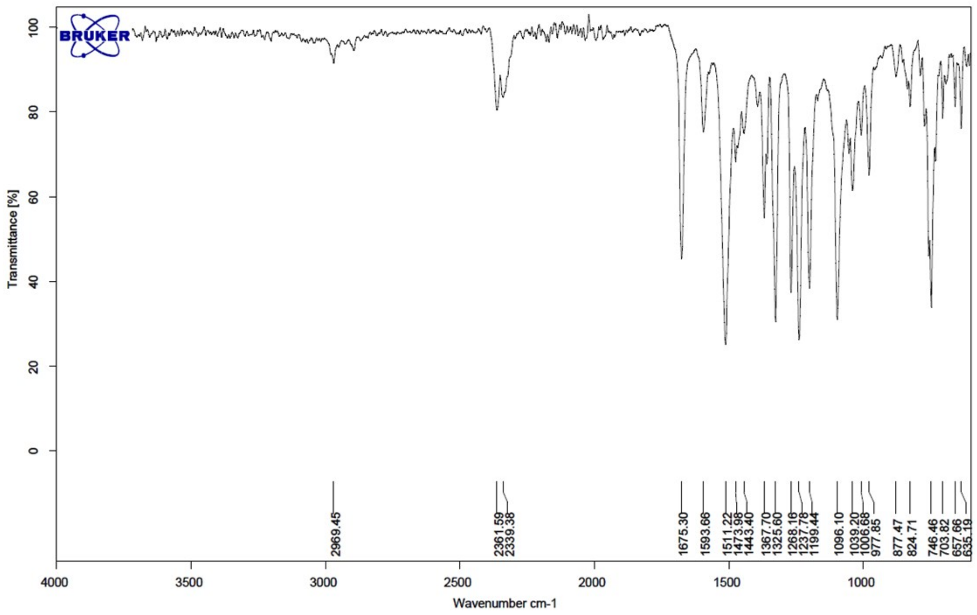

The FT-IR spectrum of product (IV_g)

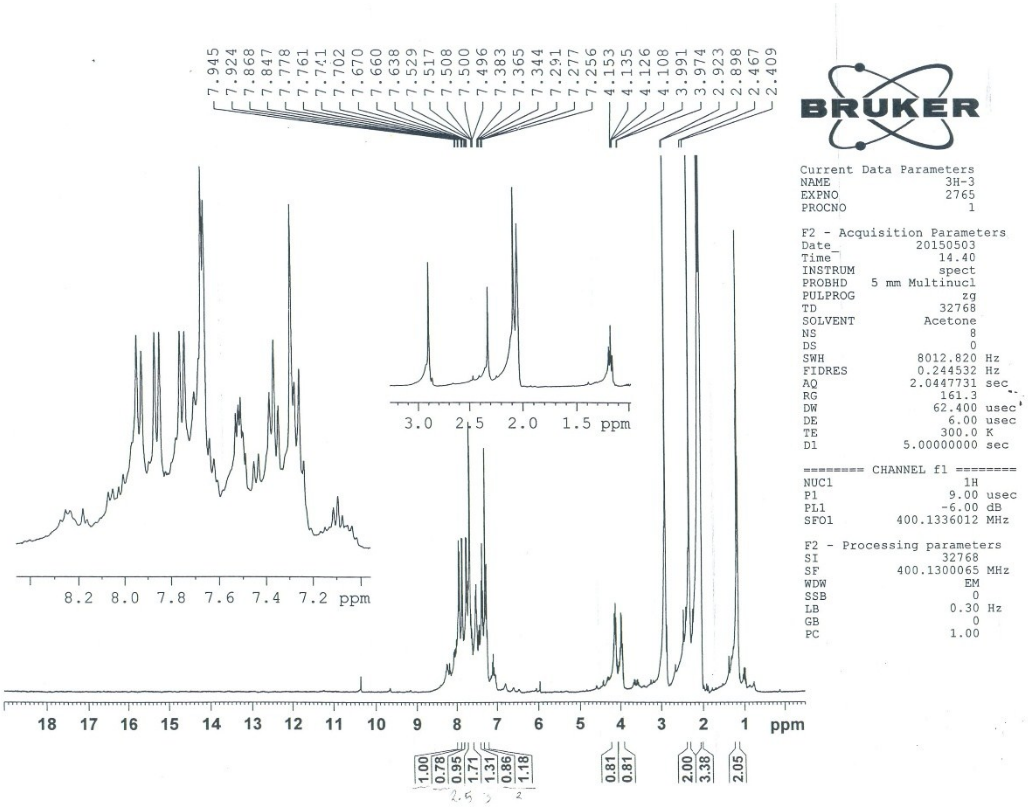

The ^{1}H NMR (400MHz) spectrum of product (IV_g)

Ethyl-2-methyl-4-(2-ethoxy phenyl)-4*H*-pyrimido[2,1-*b*][1,3]benzothiazole-3-carboxylate (table 5, IV_h).

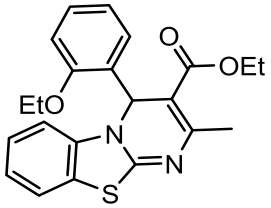

Yellow solid. ^{1}H NMR (Acetone-d$_6$, 400 MHz): δ 7.63 (d, *J*=8 Hz, 1H), 7.53 (dd, *J*=7.6, 1.6 Hz, 1H), 7.47 (d, *J*=8 Hz, 1H), 7.29 (m, 1H), 7.15-7.18 (m, 2H), 6.88-6.92 (m, 2H), 6.75 (s, 1H), 4.10-4.14 (m, 2H), 4.03 (q, *J*=7.2 Hz, 2H), 2.37 (s, 3H), 1.50-1.54 (m, 3H), 1.16-1.20 (m, 3H). IR (KBr): 2980, 1692, 1600, 1511, 1493, 1242, 1201, 1075, 1040, 752 cm^{-1}. mp: 171-175 ˚C.

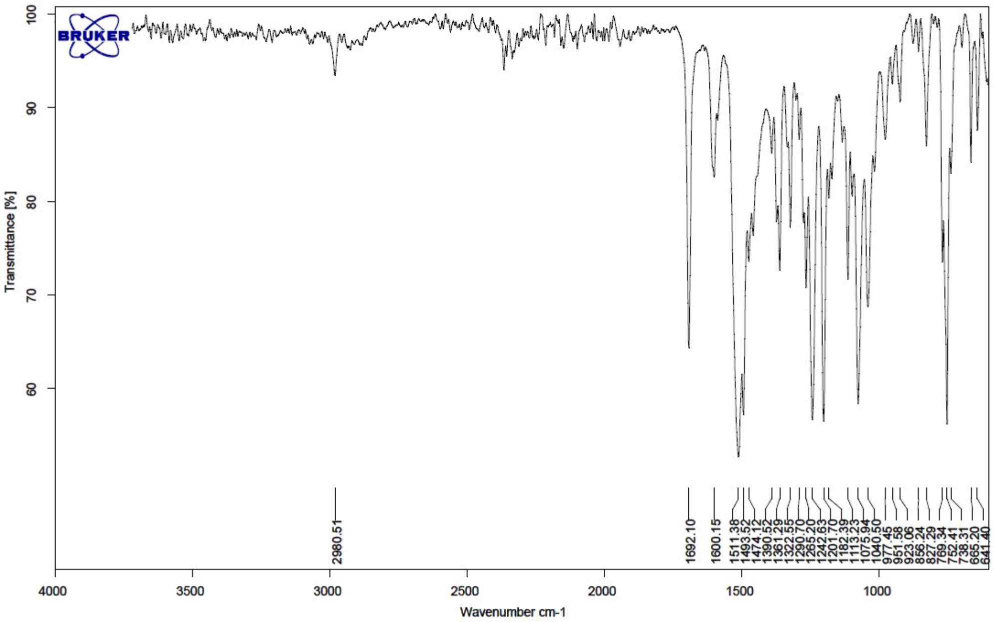

The FT-IR spectrum of product (IV_h)

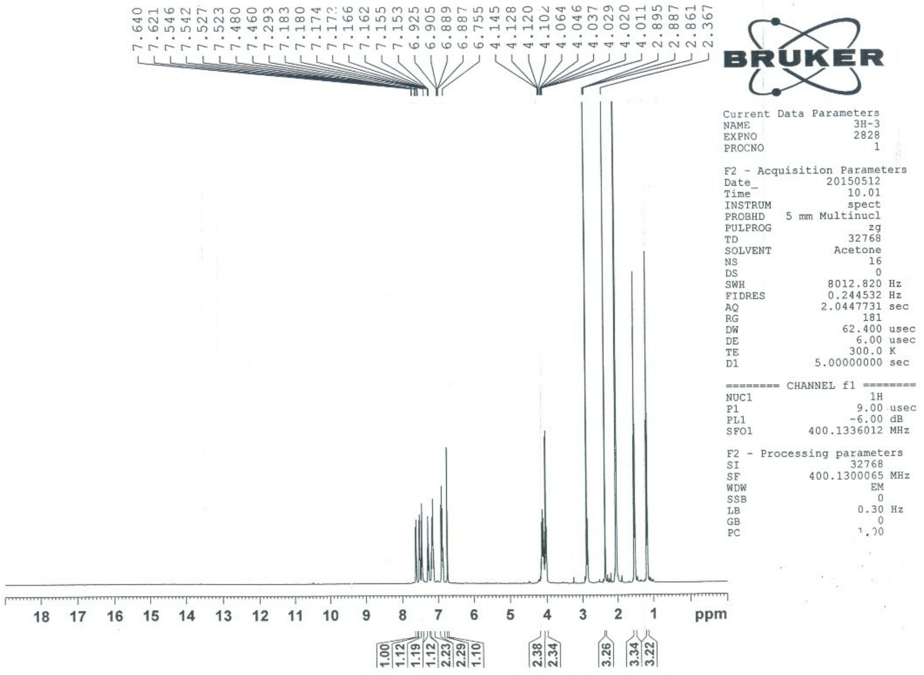

The ^{1}H NMR (400MHz) spectrum of product (IV_h)

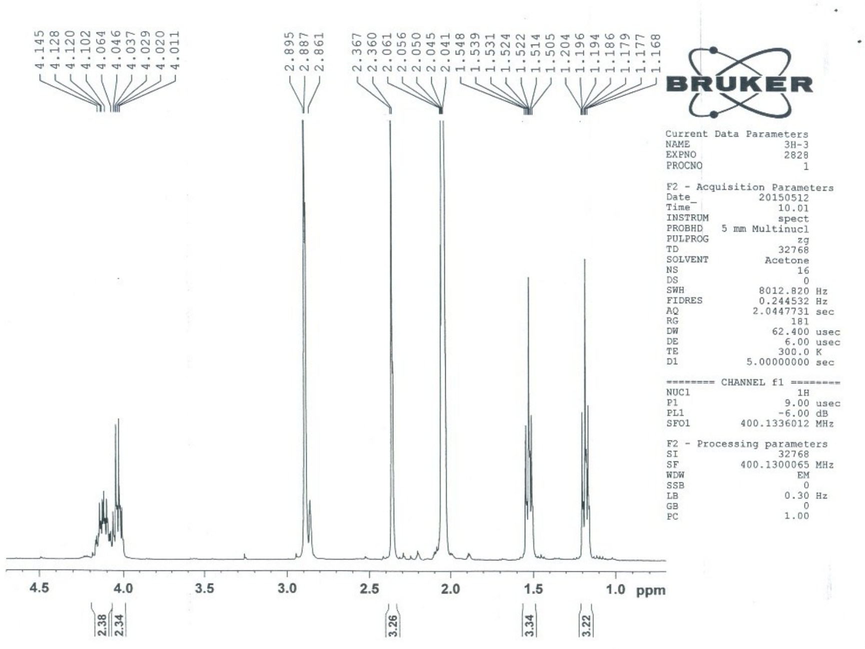

The ^{1}H NMR (400MHz) spectrum of product (IV_h)

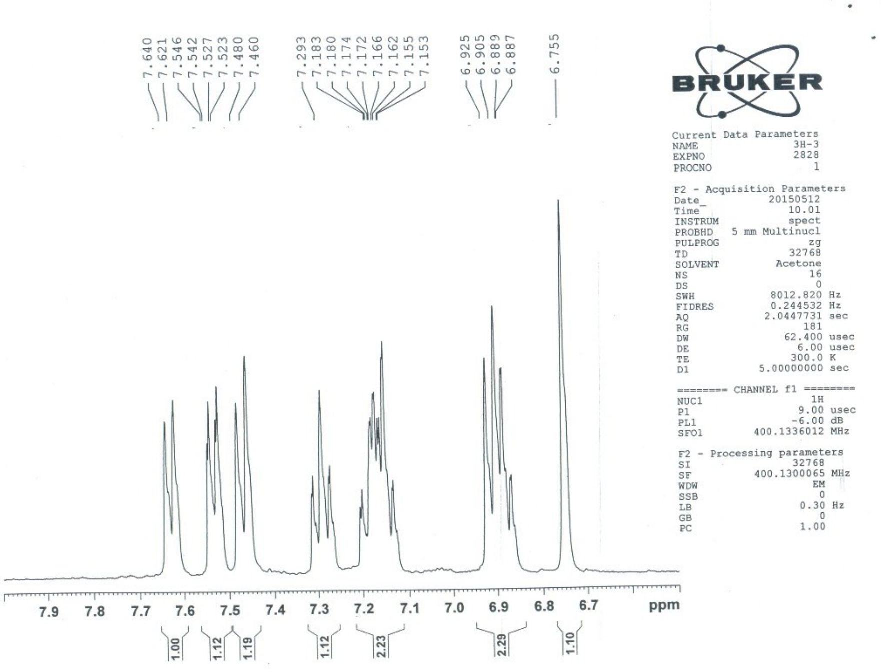

The ^{1}H NMR (400MHz) spectrum of product (IV_h)

Ethyl-2-methyl-4-(3-nitrophenyl)-4*H*-pyrimido[2,1-*b*][1,3]benzothiazole-3-carboxylate (table 5, IV_i).

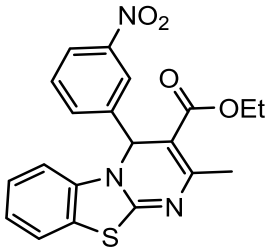

Light yellow solid. ^{1}H NMR (Acetone-d$_6$, 400 MHz): δ 8.45 (s, 1H), 8.13 (d, *J*=8 Hz, 1H), 7.91 (d, *J*=8 Hz, 1H), 7.71 (d, J=8 Hz, 1H), 7.65 (t, *J*=8 Hz, 1H), 7.47 (d, *J*=8 Hz, *1H*), 7.33 (t, *J*=8 Hz, *1H*), 7.25 (t, *J*=8 Hz, 1H), 6.74 (s, 1H), 3.90-4.20 (m, 2H), 2.30 (s, 3H), 1.30 (m, 3H). IR (KBr): 1654, 1581, 1500, 1343, 1273, 1247, 1205, 1098, 748 cm^{-1}. mp: 222-224 °C.

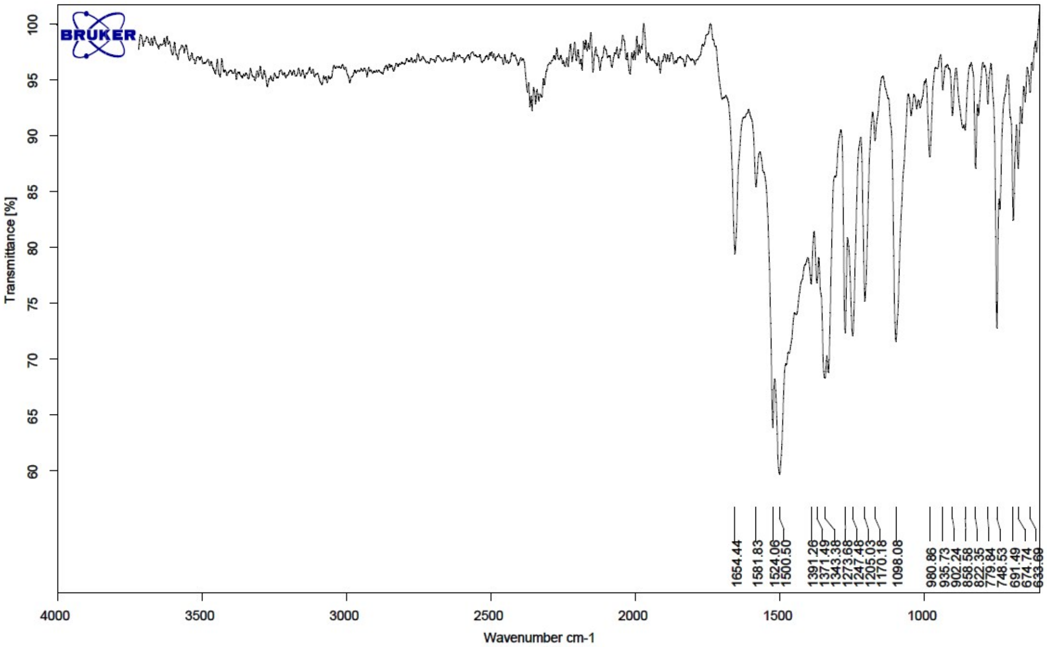

The FT-IR spectrum of product (IV_i)

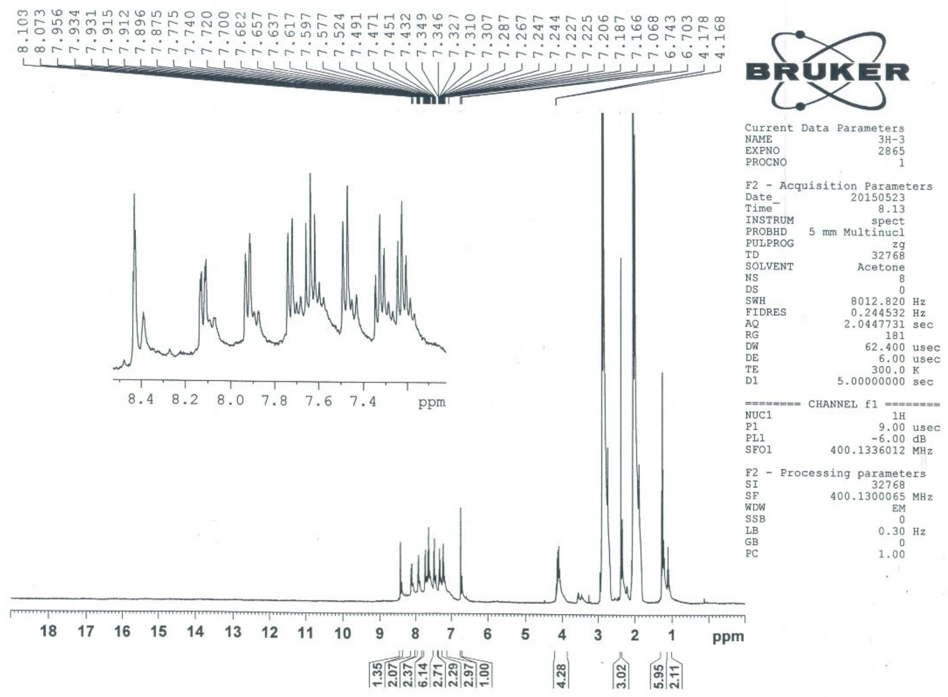

The ^{1}H NMR (400MHz) spectrum of product (IV_i)

Ethyl-2-methyl-4-(3-hydroxy phenyl)-4*H*-pyrimido[2,1-*b*][1,3]benzothiazole-3-carboxylate (table 5, IV_j).

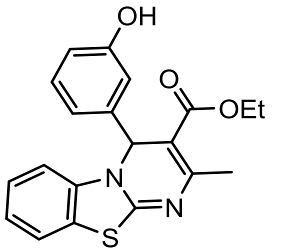

Yellow solid. ^{1}H NMR (Acetone-d$_6$, 400 MHz): δ 8.50 (s, 1H), 7.70 (m, 1H), 7.35 (m, 2H), 7.20 (m, 1H), 7.10 (m, 1H), 6.94 (m, 2H), 6.7 (m, 1H), 6.45 (s, 1H), 4.10 (q, *J*=7 Hz, 2H), 2.33 (s, 3H), 1.27 (t, *J*=7 Hz, 3H). IR (KBr): 3227, 3063, 2980, 2925, 1687, 1600, 1511, 1458, 1275, 1243, 1213, 1100, 788 cm^{-1}. mp: 260-263 °C.

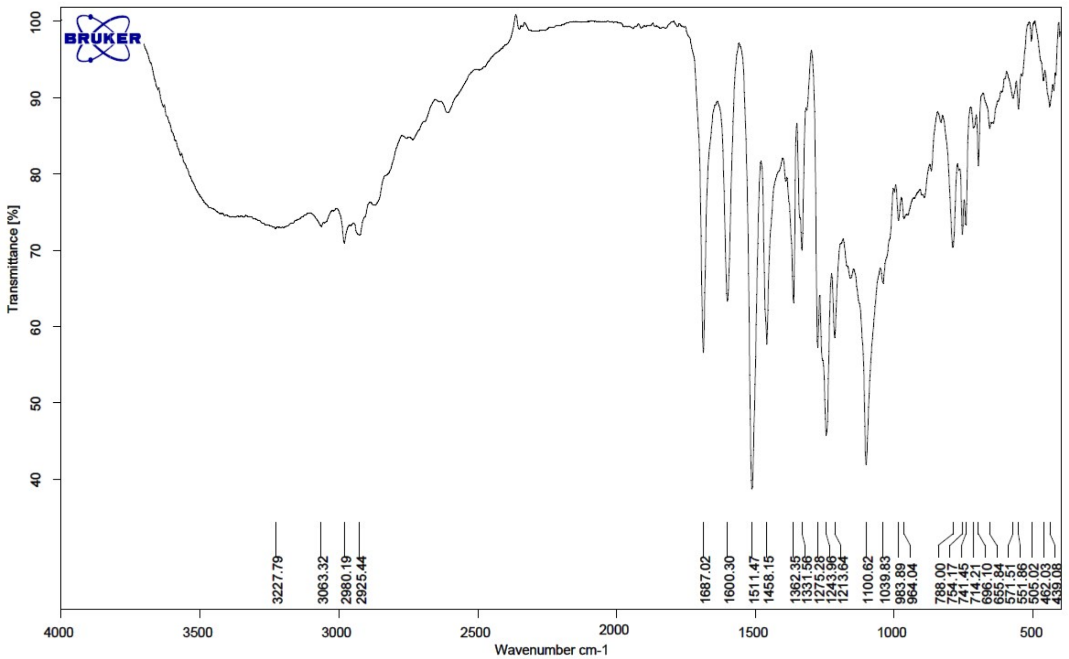

The FT-IR spectrum of product (IV_j)

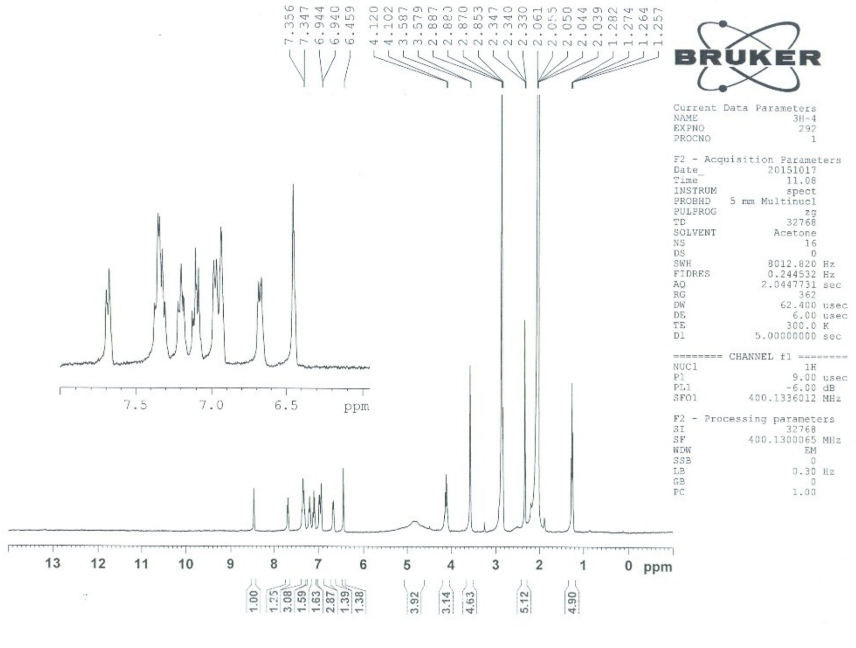

The ^{1}H NMR (400MHz) spectrum of product (IV_j)

Ethyl-2-methyl-4-(2,4-dichlorophenyl)-4*H*-pyrimido[2,1-*b*][1,3]benzothiazole-3-carboxylate (table 5, IV$_k$).

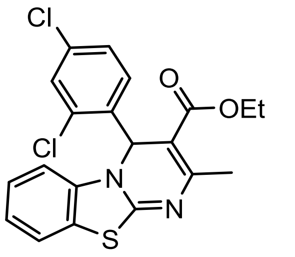

Yellow solid. ^{1}H NMR (Acetone-d$_6$, 400 MHz): δ 7.69-7.72 (m, 2H), 7.33-7.46 (m, 4H), 7.22 (t, *J*=7.6 Hz, 1H), 6.82 (s, 1H), 4.11 (q, *J*=6.8 Hz, 2H), 2.37 (s, 3H), 1.20 (t, *J*=6.8 Hz, 3H). IR (KBr): 3007, 2971, 1698, 1583, 1490, 1360, 1242, 1201, 1076, 845, 743 cm^{-1}. mp: 133-135 °C.

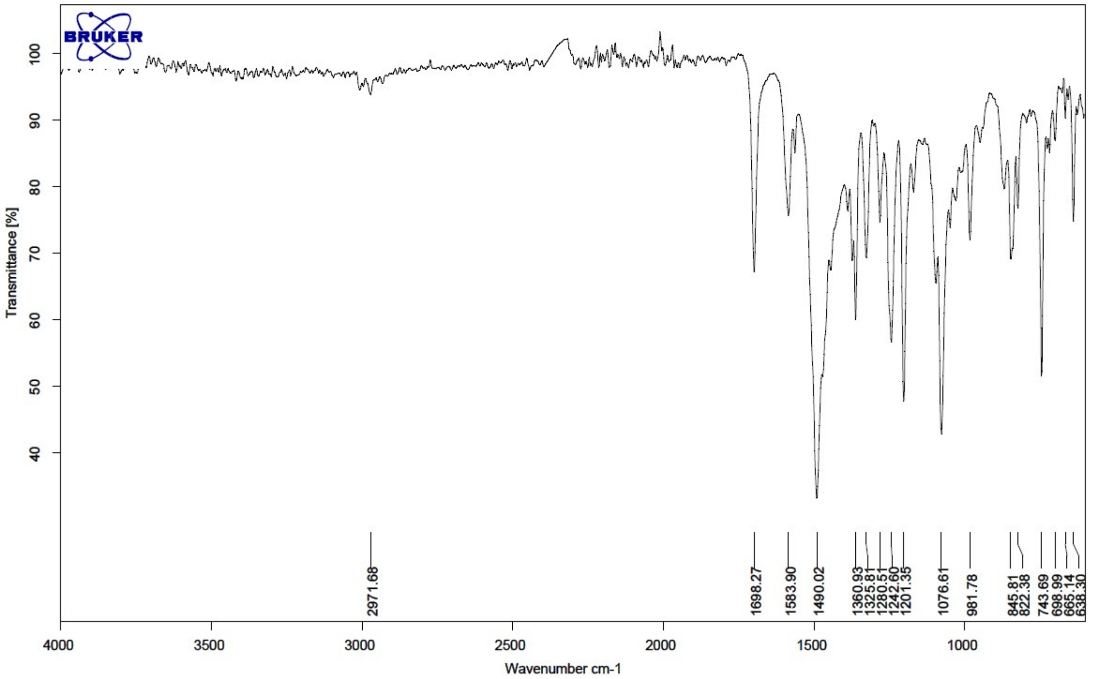

The FT-IR spectrum of product (IV$_k$)

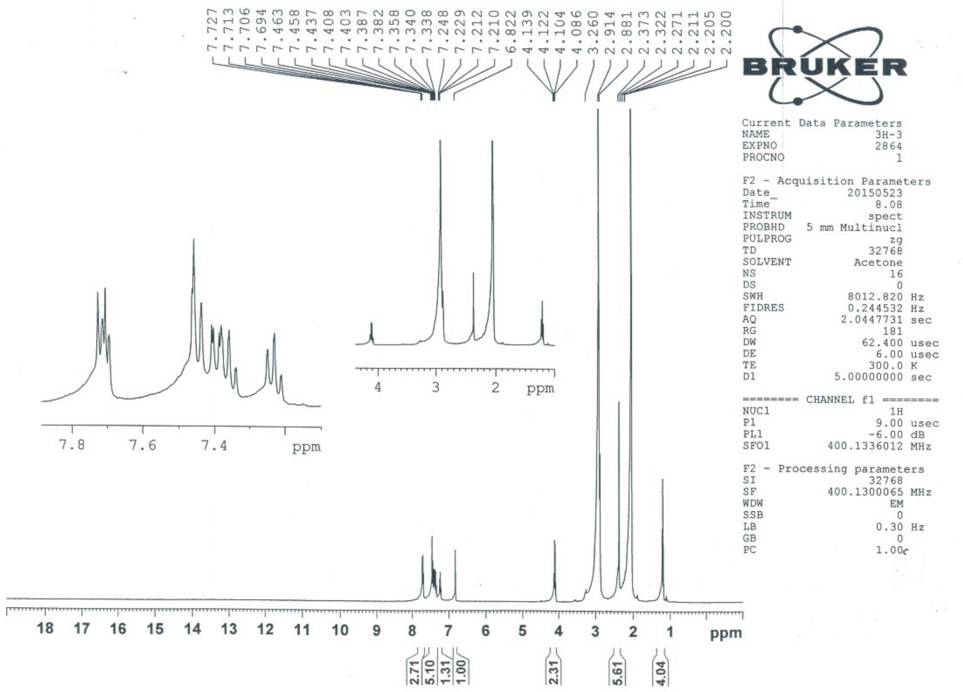

The ^{1}H NMR (400MHz) spectrum of product (IV_k)

Ethyl-2-methyl-4-(2,4-dimethoxyphenyl)-4*H*-pyrimido[2,1-*b*][1,3]benzothiazole-3-carboxylate (table 5, IV_l).

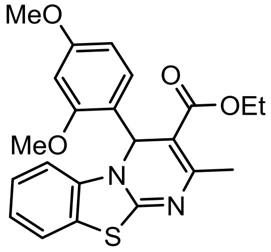

Yellow solid. ^{1}H NMR (Acetone-d$_6$, 400 MHz): δ 7.62 (d, *J*=7.6 Hz, 1H), 7.45 (d, *J*=8 Hz, 1H), 7.37 (d, *J*=8.4 Hz, 1H), 7.30 (td, *J*=8, 1.2 Hz, 1H), 7.15 (td, *J*=7.6, 1.2 Hz, 1H), 6.68 (s, 1H), 6.44-6.48 (m, 2H), 4.05 (q, *J*=6.8 Hz, 2H), 3.93 (s, 3H), 3.71 (s, 3H), 2.36 (s, 3H), 1.19 (t, *J*=6.8 Hz, 3H). IR (KBr): 1694, 1583, 1497, 1271, 1239, 1203, 1075, 836, 739 cm^{-1}. mp: 164-166 ˚C.

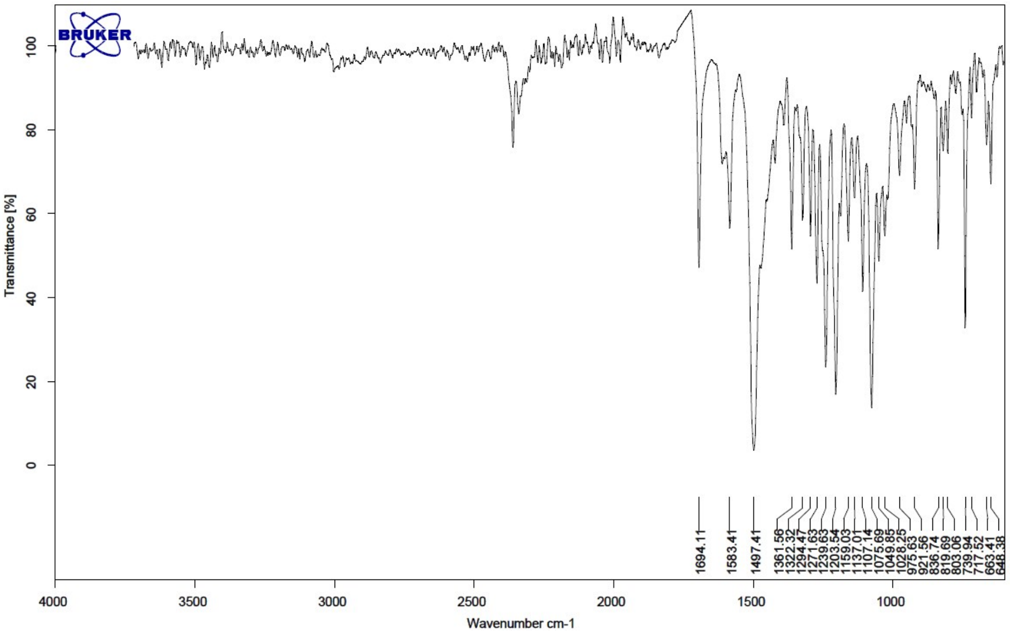

The FT-IR spectrum of product (IV_1)

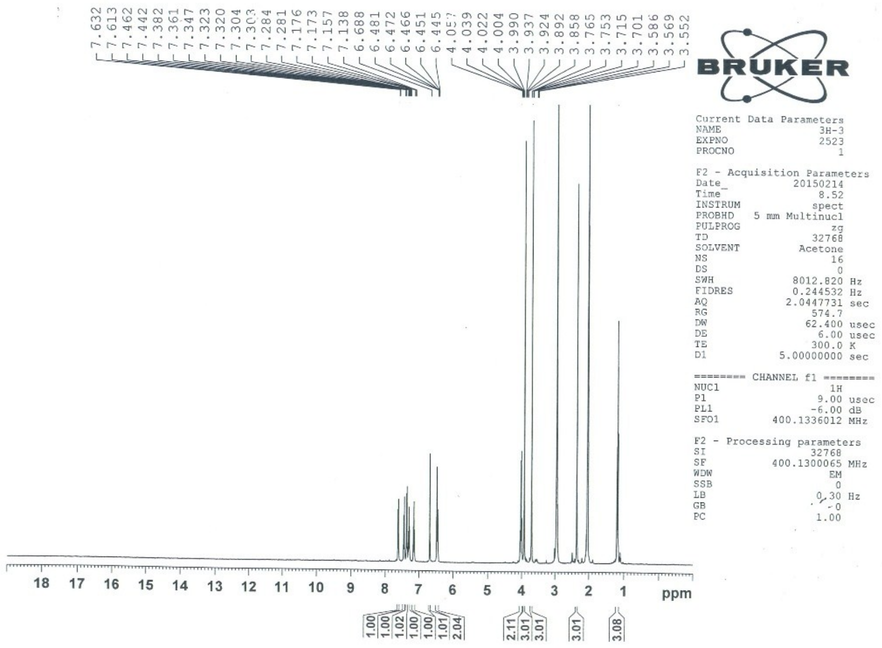

The ^{1}H NMR (400MHz) spectrum of product (IV_1)

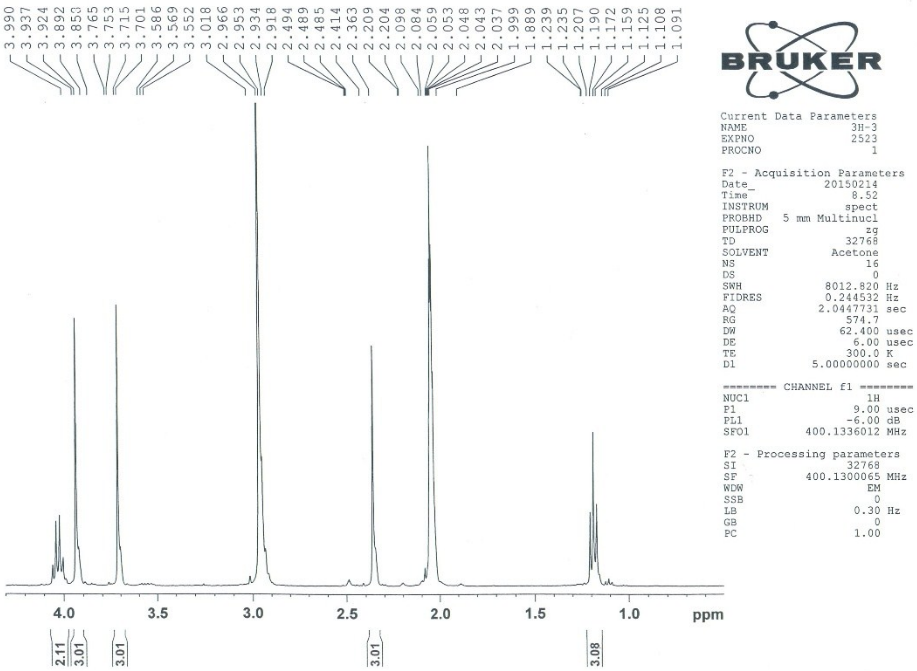

The ^{1}H NMR (400MHz) spectrum of product (IV_l)

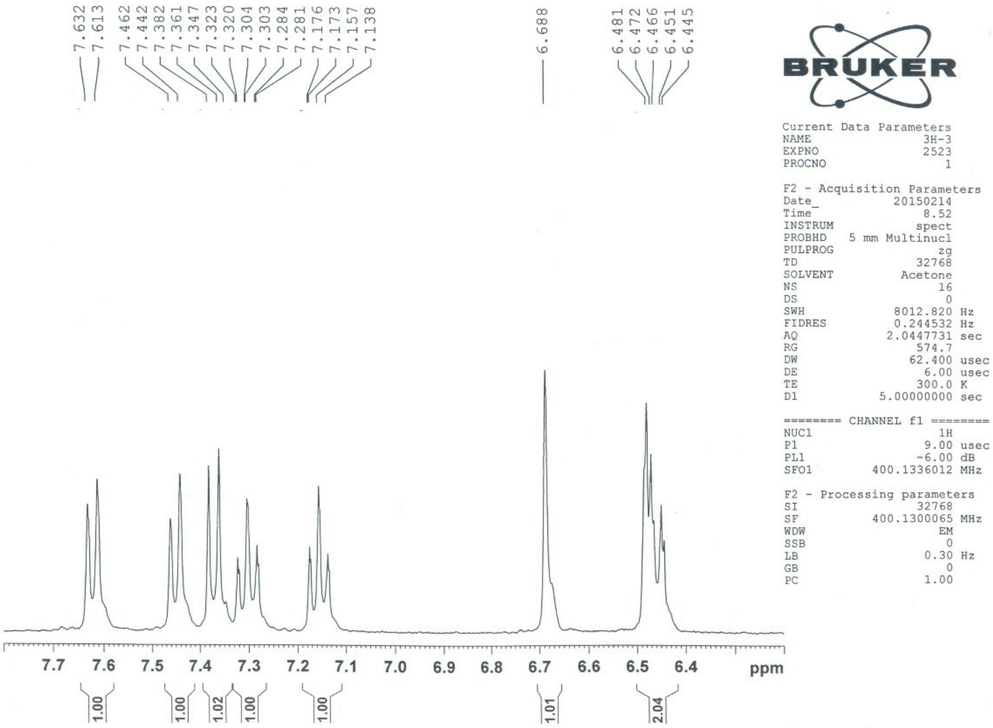

The ^{1}H NMR (400MHz) spectrum of product (IV_l)

Ethyl-2-methyl-4-(3,4-dihydroxyphenyl)-4*H*-pyrimido[2,1-*b*][1,3]benzothiazole-3-carboxylate (table 5, IV$_{m}$).

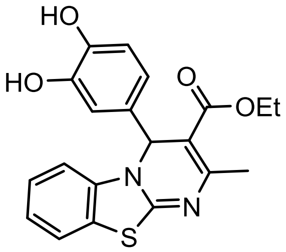

Gray solid. ^{1}H NMR (Acetone-d$_6$, 400 MHz): δ 8.30 (s, 1H), 7.91 (s, 1H), 7.64 (d, *J*=7.6 Hz, 1H), 7.37 (d, *J*=8 Hz, 1H), 7.32 (t, *J*=7.6 Hz, 1H), 7.19 (t, *J*=7.6 Hz, 1H), 6.94 (s, 1H), 6.87 (d, *J*=8 Hz, 1H), 6.71 (d, *J*=8 Hz, 1H), 6.37 (s, 1H), 4.12 (q, *J*=7.2 Hz, 2H), 2.33 (s, 3H), 1.20 (t, *J*=7.2 Hz, 3H). IR (KBr): 3380, 2981, 1661, 1595, 1507, 1446, 1274, 1252, 1211, 1100, 753 cm^{-1}. mp: 225-227 ˚C.

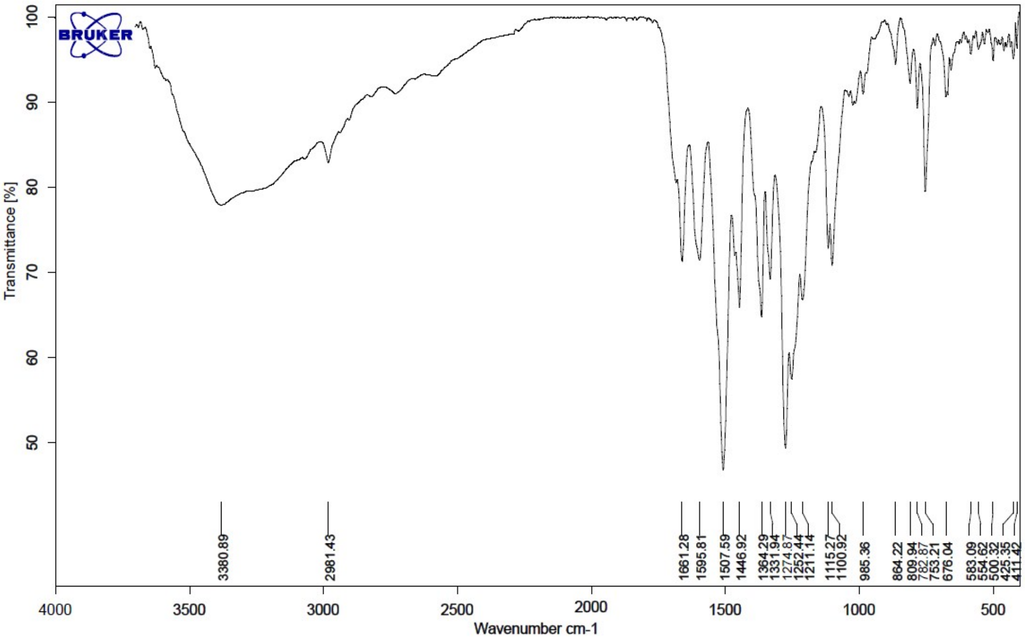

The FT-IR spectrum of product (IV$_{m}$)

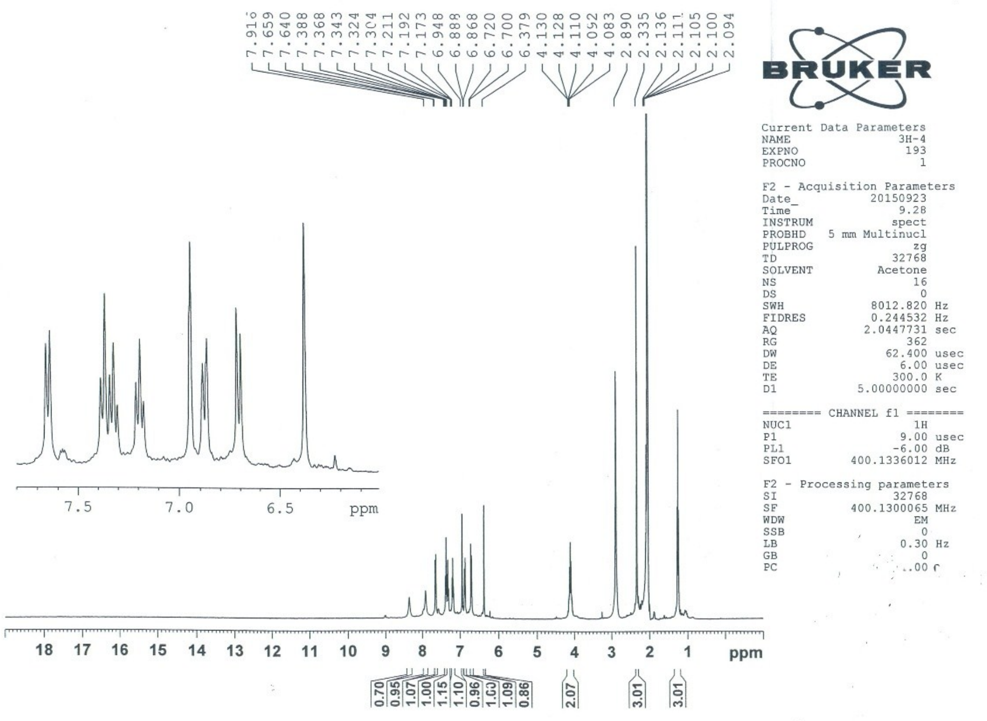

The ^1H NMR (400MHz) spectrum of product (IV_m)
